# Prevalence of daily fruit and vegetable intake by socio-economic characteristics, women’s empowerment, and climate zone: an ecological study in Latin American cities

**DOI:** 10.1017/jns.2024.93

**Published:** 2025-01-15

**Authors:** Giovanna Valentino, Amy H. Auchincloss, Binod Acharya, Natalia Tumas, Nancy López-Olmedo, Ana Ortigoza, Mariana Carvalho de Menezes, María Fernanda Kroker-Lobos, Carolina Nazzal

**Affiliations:** 1 Programa de Doctorado, Escuela de Salud Pública, Facultad de Medicina, Universidad de Chile, Santiago, Chile; 2 Departamento de Nutrición y Dietética, Escuela de Ciencias de la Salud, Facultad de Medicina, Pontificia Universidad Católica de Chile, Santiago, Chile; 3 Department of Epidemiology and Biostatistics, Dornsife School of Public Health, Drexel University, Philadelphia, PA, USA; 4 Urban Health Collaborative, Dornsife School of Public Health, Drexel University, Philadelphia, PA, USA; 5 Centro de Investigaciones y Estudios sobre Cultura y Sociedad, Consejo Nacional de Investigaciones Científicas y Técnicas (CONICET) y Universidad Nacional de Córdoba, Córdoba, Argentina; 6 Facultad de Ciencias Médicas, Universidad Nacional de Córdoba, Córdoba, Argentina; 7 Center for Population Health Research, National Institute of Public Health, Cuernavaca, Mexico; 8 Department of Social and Environmental Determinants for Heath Equity, Pan American Health Organization, Washington, DC, USA; 9 Department of Nutrition, Federal University of Minas Gerais, Belo Horizonte, Brazil; 10 INCAP Research Center for the Prevention of Chronic Diseases, Institute of Nutrition of Central America and Panama, Guatemala City, Guatemala; 11 Escuela de Salud Pública, Facultad de Medicina, Universidad de Chile, Santiago, Chile

**Keywords:** Climate zone, Diet quality, Latin America, Middle-income countries, Socio-economic development, Women’s empowerment, F&V, Fruits and vegetables, GDP, gross domestic product, WA, Women Achievement score, GII, Gender Inequality Index, SALURBAL, Salud Urbana en América Latina, ICC, intraclass correlation coefficient, CI, confidence interval, T, tertile, IQR, interquartile range

## Abstract

This cross-sectional ecological study described fruit and vegetable (F&V) intake variability across 144 cities in 8 Latin American countries and by city-level contextual variables. Data sources came from health surveys and census data (Argentina, Brazil, Chile, Colombia, El Salvador, Guatemala, Mexico, and Peru). Self-reported frequency of F&V intake was harmonised across surveys. Daily F&V intake was considered as consumption 7 d of the week. Using a mixed-effects model, we estimated age and sex-standardised city prevalences of daily F&V intake. Through Kruskal–Wallis tests, we compared city F&V daily intake prevalence by tertiles of city variables related to women’s empowerment, socio-economics, and climate zones. The median prevalence for daily F&V intake was 55.7% across all cities (22.1% to 85.4%). Compared to the least favourable tertile of city conditions, F&V daily intake prevalence was higher for cities within the most favourable tertile of per capita GDP (median = 65.7% vs. 53.0%), labour force participation (median = 68.7% vs. 49.4%), women achievement-labour force score (median = 63.9% vs. 45.7%), and gender inequality index (median = 58.6% vs. 48.6%). Also, prevalences were higher for temperate climate zones than arid climate zones (median = 65.9% vs. 50.6%). No patterns were found by city level of educational attainment, city size, or population density. This study provides evidence that the prevalence of daily F&V intake varies across Latin American cities and may be favoured by higher socio-economic development, women’s empowerment, and temperate weather. Interventions to improve F&V intake in Latin America should consider the behaviour disparities related to underlying local social, economic, and climate zone characteristics.

## Graphical abstract

**Figure:**
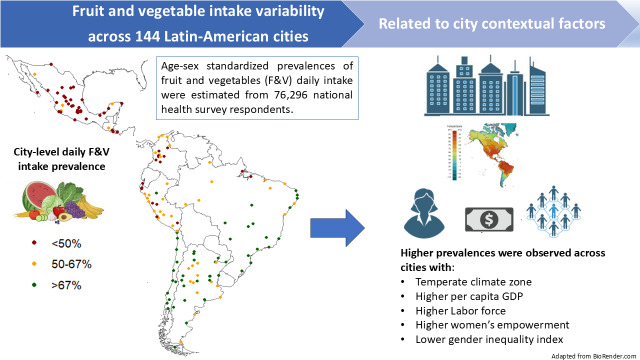

## Introduction

Epidemiological evidence shows that dietary patterns such as the Mediterranean, DASH (Dietary Approaches to Stop Hypertension), Nordic, Japanese, vegetarian, and pescatarian diets reduce the risk of morbidity and mortality from cardiovascular disease and some types of cancer^(1–3)^. The main shared characteristic of these dietary patterns is the high consumption of unprocessed plant-based foods, especially fruits and vegetables (F&V). Although the Latin American region is a major producer of fresh F&V^(4)^, studies show that compliance with a healthy diet is low in Latin American countries: only 4% to 15% of people meet F&V intake recommendations of ≥5 servings/d^(5)^.

Prior work in the Latin American region at the country level has found higher GDP, lower unemployment, and greater urbanicity linked to higher country-level consumption of F&V^(6,7)^. Other work focused on cities in Belo Horizonte and Campinas (Brazil) and Mexico City (Mexico), have found that access to local fresh products markets is linked to higher consumption of F&V^(8–11)^. The study of the patterns of F&V intake and their determinants in cities is relevant since approximately 56% of the world’s population lives in cities, and Latin America is the most urbanised region in the world (∼80% of the total population)^(12)^. However, limited research has described the variability in F&V consumption between cities according to city characteristics. Even within a country, between-city variability may be substantial in Latin American cities, given the region’s high social inequality. For example, data from the Chilean National Health Survey 2016-2017 shows that, although overall F&V intake at the national level is low (∼15% meet guidelines), there is great heterogeneity across regions, ranging from 6% (in the southern region of Aisen) to 26% (in the central region of Valparaiso)^(13)^.

Several factors could underlie local variability in nutritional quality and food systems, including F&V supply and population intake^(14)^. The UNICEF *Innocenti Framework* articulates how food systems shape children’s diet and identifies key drivers of food systems, which also apply to adults´ diet: 1) economic and social drivers (such as employment, GDP, inequality/equality [including gender equality] and social inclusion), 2) demographic drivers (such as urbanisation and population size), and 3) biophysical drivers (such as climate suitability)^(14,15)^. Similar to previous country-level evidence^(6,7)^, cities with less favourable *economic and social context* (e.g. lower subnational GDP and labour force participation) could have lower F&V consumption, which may be explained by inadequate supply and lower purchasing power. Also, cities with higher educational attainment may have higher consumption, as individual educational level is a strong determinant of diet^(5)^. Women’s empowerment has also shown a link with better nutritional status and diet quality in studies in low- and middle-income countries^(16,17)^. The latter might be explained by a higher decision-making power of women, primarily through roles such as caretaking and food purchases for cooking. In addition, demographic characteristics, including greater city population size and high density, could improve access to food distribution centres and influence purchasing and consumption patterns^(18)^. Climate zones and climate change are closely related to F&V production, which may significantly influence supply and population dietary behaviours^(19)^.

The present ecological study aims to describe the variability of F&V consumption across cities in 8 Latin American countries and by city-level contextual variables (socio-economic, demographic, and climate zone variables). We hypothesise that cities with more favourable socio-economic characteristics (e.g. higher GDP per capita, higher employment, higher education attainment), women’s empowerment, and higher population size and density will have higher prevalences of F&V daily intake and that cities with lower prevalences of F&V daily intake will belong to extreme climate zones (e.g. Arid) as these could affect local F&V production, supply and affordability.

## Methods

### Study design and sample

This cross-sectional ecological study, utilised secondary data compiled as part of the SALURBAL project (SaLud Urbana en América Latina—Urban Health in Latin America). SALURBAL compiled and harmonised databases of national health surveys, censuses, and other surveys from 371 cities in 11 Latin American countries to examine differences in health between and within urban areas. SALURBAL selected cities with urban agglomerations of ≥100,000 inhabitants, and variables from the datasets were merged by a geographic crosswalk defined by the city boundaries (described in detail elsewhere^(20)^). The SALURBAL protocol was conducted according to the guidelines laid down in the Declaration of Helsinki and all procedures involving research study participants were approved by the Drexel University Institutional Review Board with ID #1612005035 and by appropriate site-specific IRBs.

Dietary data were available from health surveys in a subset of SALURBAL cities: 234 cities in 8 countries (Argentina, 2013; Brazil, 2013; Chile, 2017; Colombia, 2015; El Salvador, 2015; Guatemala, 2002; Mexico, 2018; and Peru, 2016). To have a sufficient sample size to assess city-level prevalences, we restricted our analysis to cities with a minimum of 100 individuals aged 20–69 years old surveyed, resulting in a final sample of 76,726 individuals from 144 cities (further details in Supplementary Material, Figure S1, and Table S2).

### Variables

#### City prevalence of fruit and vegetable daily intake

Survey questions regarding self-reported fruit and vegetable consumption were collected from health surveys. Most surveys asked for weekly consumption of each group (fruits and vegetables), which were harmonised. For Mexico, which used a food frequency questionnaire (FFQ), the most frequent fruit and vegetable was considered to avoid overestimation of intake. Supplementary Material describes the harmonised data, sample design, and methods for each survey (Section 1.1, Table S1, Table S2). The frequency of fruit and vegetable intake (days/week) was summed to classify whether fruits and vegetables were consumed 7 d a week (‘daily F&V’ intake) and then a binary variable was created. Then, we estimated the standardised city-level prevalence of daily F&V consumption. We first applied a country-stratified mixed-effect logistic regression model. Random intercepts were used for the cities, and fixed effects were included for age, sex, and education. Subsequently, we standardised our estimates of daily F&V using the age (20-69 years old) and sex distribution of the population for all the cities combined (to make estimates comparable across cities). The standardisation/post-stratification weights were calculated using the population projections for the year 2010. This approach using multilevel modelling yields estimates that are stable and more precise (for further details, see Supplementary Material, section 1.2).

#### Socio-economic factors

We included census data for city-level socio-economic variables from the closest available year to the health survey data of each country (Table S3). We considered the following socio-economic variables: per capita ***Gross Domestic Product (GDP), labour force participation, and educational attainment***. City-level per capita GDP (expressed in constant 2011 USD) was obtained from a gridded global dataset, which estimated subnational GDP at the smallest administrative unit possible using complete data from a range of government, survey, and industry sources^(21,22)^. Subnational GDP estimates were available for 1990–2015 and assigned to cities located within the corresponding administrative unit. We used the GDP per capita from the same year in which dietary data was collected or the closest year available (2015) if the health survey was posterior to 2015. The labour force participation rate and the proportion of the population aged ≥15 years old working or actively seeking work were obtained from census data. City educational attainment considered the proportion of the population aged ≥25 who completed high school. Both census variables were defined according to IPUMS (Integrated Public Use Microdata Series) criteria for harmonisation (Table S3).

#### Women’s empowerment

Women’s empowerment was proxied by ***women’s achievement score in the labour force (WA-Labor force)*** and ***women’s achievement score in education (WA-Education),*** which included variables identified through factor analysis as previously described^(23)^. WA-Labor force is related to women’s autonomy and includes female labour force participation and formal marriage in women between 15 and 17 years old. WA-Education included the proportion of women ≥25 years old with completed secondary education and the proportion of women with completed higher education. Both were expressed as a Z-score based on the distribution of scores across all SALURBAL cities, with higher values indicating higher achievements. ***Gender inequality index (GII)*** was adapted from the index developed by the United Nations Development Fund, which uses country-level data^(23)^. As described elsewhere^(23)^, it included male versus female labour force participation, high school attainment, and government representation based on the percentage of mayoral positions occupied by women and men (Table S3). The GII ranged from 0 to 1, with values closer to 1 indicating higher inequality.

#### Urban demographic factors

***City size*** was determined using census data for the year closest to the country’s national health survey. It was defined as the population projection residing in the city administrative area the same year of the national health survey^(20)^. ***Population density*** was defined as inhabitants by squared kilometres of the built-up area calculated using the total population for the city divided by the administrative area. Both variables were treated as continuous variables^(20)^ (Table S3).

#### Climate zone

The ***major climate zone*** of each city was classified using the Köppen system, which considers four main categories: tropical, arid, temperate, and polar (Table S3)^(24,25)^. The tropical climate has an average temperature of ≥18°C every month and has a high precipitation rate (including tropical rainforest, savannah, and monsoon climate)^(24)^. Arid climates are defined by a low precipitation rate in regions that cannot be classified as polar (including hot and cold deserts and semi-arid climates)^(24)^. Temperate climate includes regions where the coldest month has an average temperature between -3°C and 18°C and at least one month averaging >10°C (includes subtropical, subpolar, and Mediterranean climates)^(24)^. Polar climate includes regions with no month averaging temperature ≥10°C^(24)^.

### Statistical analysis

To describe the city characteristics for each country, we reported city-level F&V daily intake prevalences and contextual variables as medians and interquartile range. Climate zone categories were reported as absolute and relative frequencies. Individual characteristics of survey participants can be found in Supplementary Material (Table S4).

The distribution of city-level F&V daily intake prevalences was described by country, tertiles of socio-economic, women’s empowerment, demographic factors, and climate zone. Kruskal–Wallis test with Dunn’s test was performed to compare the median of city prevalences across tertiles. The heterogeneity of cities’ prevalences for F&V daily intake was determined by estimating the intraclass correlation coefficient (ICC) in a two-level linear regression null model with city prevalence as a continuous outcome and countries as a random intercept. All the analyses were performed using Stata 18.0 and R software 4.3^(26,27)^, and we considered an alpha of 5% for statistical significance.

#### Sensitivity analyses

First, we performed additional estimations for prevalences of daily fruit intake (self-reported fruit intake of 7 d a week) and daily vegetable intake (self-reported vegetable intake of 7 d a week) separately to evaluate if patterns were consistent with the results of daily F&V intake. Also, to further assess consistency, we repeated the main analysis, excluding cities from Mexico (which used a different dietary assessment method).

## Results

**Figure 1.**
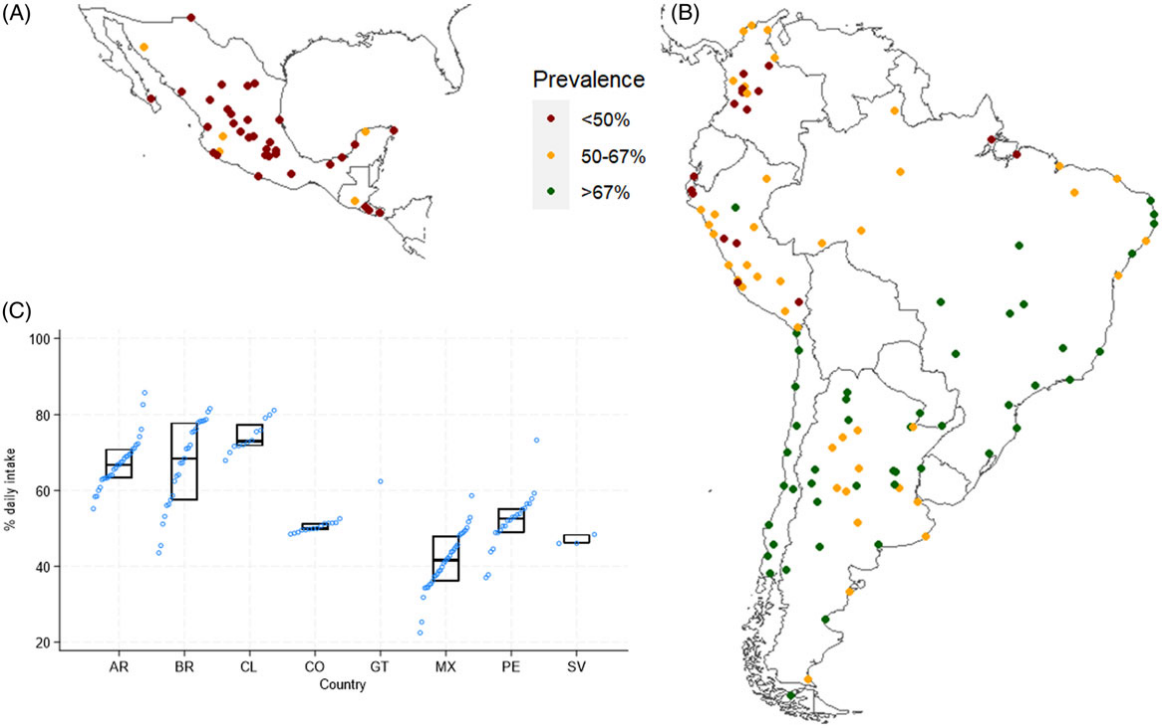
Prevalences of daily fruit and vegetable intake for 144 Latin American cities. AR, Argentina, BR, Brazil, CL, Chile, CO, Colombia, GT, Guatemala, MX, Mexico, PE, Peru, SV, El Salvador. Daily F&V intake was derived from the following health surveys: 2013 for Argentina and Brazil, 2002 for Guatemala, 2015 for Colombia and El Salvador, 2016 for Peru, 2017 for Chile, and 2018 for Mexico. Prevalences are standardised by sex and age (20-69 years old) according to the 2010 population distribution of all the cities combined. Map A shows cities from Mexico, Guatemala, and El Salvador. Map B shows Colombia, Peru, Brazil, Chile, and Argentina cities. On Panel C, each blue dot indicates a city.

The number of cities and their characteristics by country are summarised in Table 1. The country with the highest median for F&V daily consumption prevalences across cities was Chile, with 73%, followed by Brazil (68%) and Argentina (67%), whereas Mexico (42%) and El Salvador (46%) had the lowest median. Figure 1 summarises the distribution of prevalences of daily F&V intake for cities within countries, which ranged from 22.4% (Villahermosa, Mexico) to 85.8% (Formosa, Argentina). 33% of city-level F&V daily intake prevalence variability was between cities within countries, whereas 67% was between countries (ICC = 0.67; 95% CI = 0.41-0.85). A complete list of cities with their estimated prevalence, 95% confidence interval, and sample included can be retrieved from the Supplementary Material (Table S5).

**Table 1.** City socio-economic, demographic and climate zone characteristics, total and by country

	Cities for all countries (n = 144)	Argentina (n = 31)	Brazil (n = 27)	Chile (n = 12)	Colombia (n = 14)	Guatemala (n = 1)	Mexico (n = 34)	Peru (n = 22)	El Salvador (n = 3)
City prevalence of daily F&V intake, %	55.7 [20.5]	66.8 [7.6]	68.4 [20.4]	72.9 [5.8]	50.0 [1.8]	62.3	41.5 [12.0]	52.3 [6.4]	46.0 [2.3]
City prevalence of daily fruit intake, %	27.9 [14.3]	32.0 [6.3]	28.2 [10.9]	29.5 [4.0]	19.5 [7.7]	46.1	17.9 [4.6]	35.7 [6.7]	24.5 [2.4]
City prevalence of daily vegetable intake, %	28.8 [22.0]	37.3 [9.3]	36.0 [22.1]	50.0 [6.5]	29.7 [3.7]	30.5	16.9 [7.7]	14.3 [7.5]	13.1 [4.8]
Demographic characteristics									
Population, hab (per 100,000)	5.6 [8.7]	3.4 [4.2]	13.7 [26.0]	3.4 [5.3]	8.0 [15.0]	29.6	8.9 [6.8]	3.4 [2.9]	2.6 [16.6]
Population density, 1,000 hab/km^2^	7.6 [5.2]	5.2 [1.5]	7.2 [3.7]	8.7 [3.2]	17.4 [6.0]	12.3	6.6 [1.4]	11.9 [4.1]	13.5 [3.5]
Climate zone									
Tropical, n (%)	48 (33%)	0	23 (85%)	0	10 (71%)	1 (100%)	8 (24%)	3 (14%)	3 (100%)
Arid, n (%)	47 (33%)	9 (29%)	0	5 (42%)	0	0	20 (59%)	13 (59%)	0
Temperate, n (%)	47 (33%)	22 (71%)	4 (15%)	6 (50%)	4 (29%)	0	6 (18%)	5 (23%)	0
Polar, n (%)	2 (1%)	0	0	1 (8%)	0	0	0	1 (4%)	0
Socio-economic characteristics									
Per capita GDP (ppp)], USD (2011)	13,302 [11,311]	20,010 [11,506]	11,071 [12,546]	19,567 [17,526]	10,352 [3,406]	13,522	15,884 [7,264]	8,056 [7,294]	7,430 [3,243]
Labour force participation rate, %	62.7 [7.3]	66.9 [3.4]	66.7 [7.1]	59.5 [6.0]	57.7 [2.8]	59.2	61.2 [4.0]	60.8 [5.6]	57.4 [6.5]
Population with high school, %	47.9 [18.9]	37.5 [5.2]	46.3 [6.2]	64.7 [2.6]	58.3 [3.7]	26.7	47.7 [8.6]	65.9 [8.7]	26.7 [12.2]
Women’s empowerment and gender inequality									
WA-Labor Force, Z-score	0.03 [2.40]	0.86 [0.75]	1.66 [1.09]	−1.39 [0.75]	−0.76 [0.71]	0.03	−1.89 [1.15]	0.28 [1.06]	−1.4 [1.73]
WA-Education, Z-score	0.48 [2.81]	−0.70 [0.84]	0.84 [0.96]	2.66 [0.83]	2.31 [0.72]	−1.23	−0.48 [1.63]	2.99 [1.74]	−2.91 [1.54]
Gender inequality index	0.38 [0.20]	0.37 [0.29]	0.36 [0.26]	0.39 [0.20]	0.40 [0.25]	0.42	0.42 [0.21]	0.41 [0.02]	0.41 [0.28]

F&V, fruits and vegetables; GDP, gross domestic product; WA, Women Achievement; ppp, purchasing power parity. Data are expressed as median [interquartile range] or n (%). F&V data were derived for the following years of health surveys: 2013 for Argentina and Brazil, 2002 for Guatemala, 2015 for Colombia and El Salvador, 2016 for Peru, 2017 for Chile, and 2018 for Mexico.

### Prevalence of F&V daily intake by city socio-economic factors

**Figure 2.**
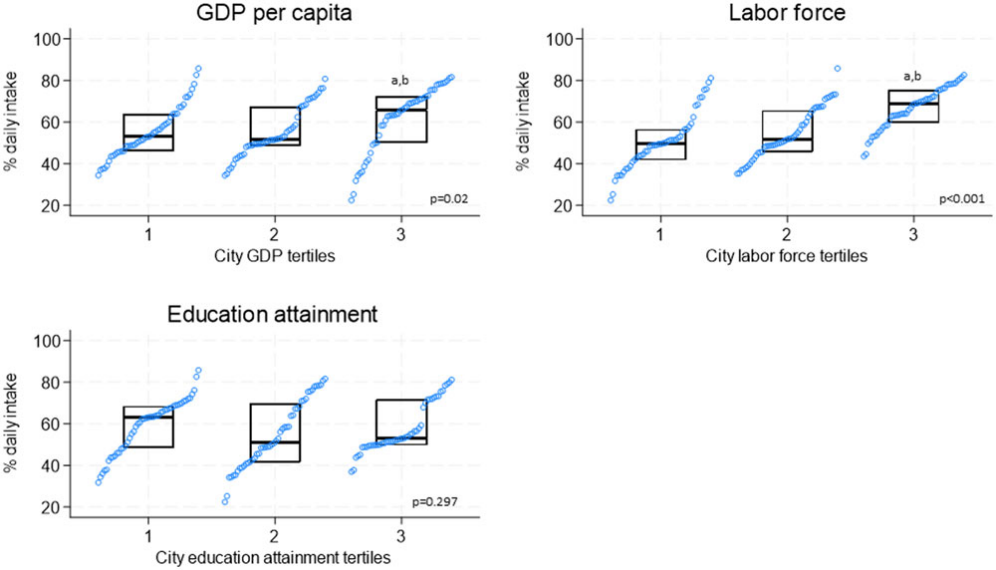
Prevalences of daily fruit and vegetable intake by tertiles of city socio-economic factors. GDP, gross domestic product. ^a^p<0.05 from tertile 1; ^b^p<0.05 from tertile 2. P value in the figure represents the p value for the trend (Kruskal–Wallis test). Prevalences are standardised by sex and age (20–69 years old) according to the 2010 population distribution of all the cities combined. GDP Tertiles: 1) <10,800 USD; 2) 10,800–17,300 USD; 3) ≥17,300 USD. City labour force tertiles:1) <60.5%; 2) 60.5% to <64.5%; 3) ≥64.6%. City education attainment tertiles (% with high school): 1)<42%; 2) 42% to <55%; 3) ≥55%. Highest tertile for GDP, labour force, and education attainment indicate cities with higher socio-economic development of the cities.

Figure 2 summarises city prevalences for daily F&V intake according to tertiles for socio-economic factors. Cities in the highest tertile (T3) of per capita GDP (≥17,300 USD) had higher prevalences of daily F&V intake compared to cities in the lowest tertile (median [IQR]: T3 = 65.7% [±22.5%] vs. T1 = 53.0% [±17.9%]). Also, cities at the highest tertile of labour force participation (≥64.5%) had higher prevalences of daily F&V intake (median [IQR]: T3 = 68.7% [±15.8%] vs. T1 = 49.4% [±14.7%]).

### Prevalence of F&V daily intake by city women’s empowerment

**Figure 3.**
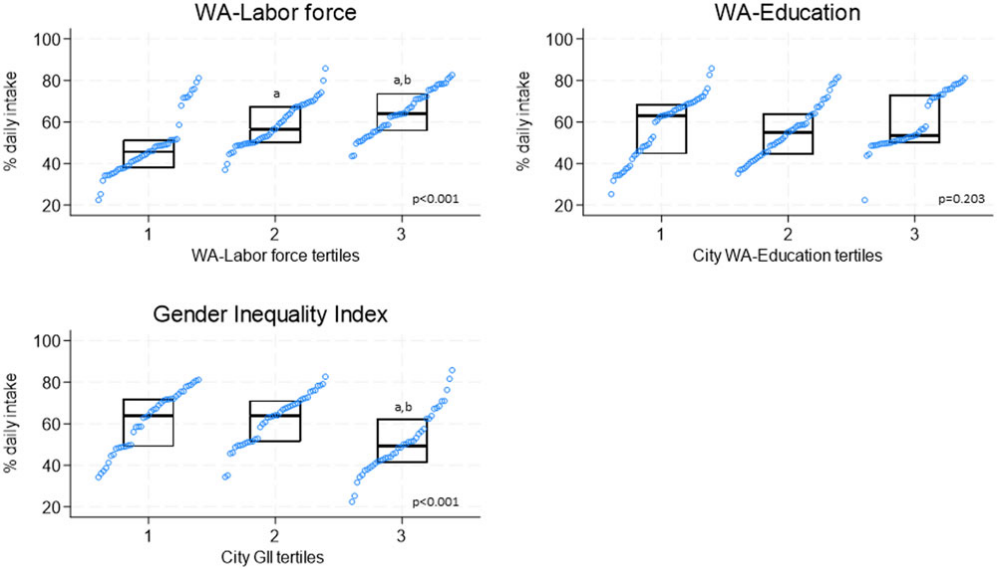
Prevalences of daily fruit and vegetable intake by tertiles of women’s empowerment. WA, Women Achievement; GII, Gender inequality index. ^a^p<0.05 from tertile 1; ^b^p<0.05 from tertile 2. P value in the figure represents the p value for the trend (Kruskal–Wallis test). Prevalences are standardised by sex and age (20–69 years old) according to the 2010 population distribution of all the cities combined. Higher labour and education WA score tertiles indicate cities with higher women autonomy (includes proportion of female labour force participation and formal marriage in women 15–17 years old) and female educational attainment scores (includes proportion women ≥25 years old with high school and college education), respectively. Higher tertile of gender inequality index (GII) indicate cities with higher gender inequality (includes labour force, political participation and educational attainment in relation to males). WA-Labor Force tertiles: 1) <-1.10 2) -1.10 to <0.85; 3) ≥0.85. WA Education tertiles: 1) <-0.35 2) -0.35 to <1.55; 3) ≥1.55. GII tertiles: 1) <0.36; 2) 0.36 to <0.40; 3) ≥0.40.

Figure 3 summarises city prevalences for daily F&V intake according to tertiles of women’s empowerment indexes. Prevalence of daily F&V intake was higher at higher levels of WA-Labor force (median [IQR]: T1 = 45.7% [±13.7%] vs. T2 = 56.5% [±17.7%] vs. T3 = 63.9% [±18.2%]) and were lower at higher levels of GII (T3) compared to other tertiles (median [IQR]: T3 = 48.6% [±13.1%] vs. T2 = 67.0% [±14.7%] vs. T1 = 58.6% [±22.6%]). In contrast, the distribution of daily F&V intake prevalences by tertiles of WA-Education did not show any clear pattern.

### Prevalence of F&V daily intake by city size, city density, and climate zone

**Figure 4.**
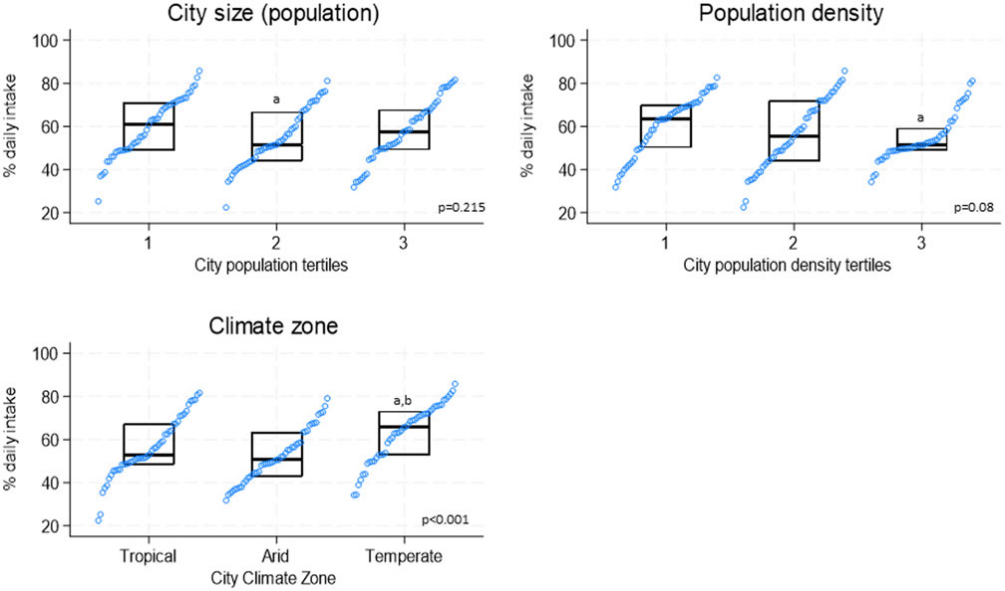
Prevalences of daily fruit and vegetable intake by tertiles of demographic factors and climate zone. ^a^p<0.05 from tertile 1 or tropical climate zone; ^b^p<0.05 from tertile 2 or arid climate zone. P value in the figure represents the p value for the trend (Kruskal–Wallis test). Prevalences are standardised by sex and age (20–69 years old) according to the 2010 population distribution of all the cities combined. City size tertiles: 1) <363,000 hab; 2) 363,000 to <1,000,000 hab; 3) ≥1,000,000 hab. City population density tertiles: 1) <6,400 hab/km^2^; 2) 6,400 to <9,600 hab/km^2^; 3) ≥9,600 hab/km^2^. Cities with polar climate zone were excluded because of sample size (n=2).

Figure 4 summarises city prevalences for daily F&V intake according to tertiles of demographic variables and climate zones. Cities at the lowest population density tertile (T1 <6,400 hab/km^2^) showed higher prevalences for F&V daily intake compared to cities in the highest tertile (median [IQR]: T1 = 63.7% [±19.9%] vs. T3 = 51.4% [±10.4%]). Also, cities in the temperate climate zone had higher prevalences compared to cities in arid or tropical zones (median [IQR]: 65.9% [±20.3%] vs. 50.6% [±20.5%] vs. 52.8% [±18.8%], respectively). Only two cities had polar climate zones, and the prevalences for daily intake were 70.0% and 49.4%.

Additionally, we performed a sensitivity analysis for fruits and vegetables separately. The median of the prevalences for daily fruit intake across cities was 27.9%, ranging from 7.1% (Villahermosa, Mexico) to 54.2% (Tarapoto, Perú). The median prevalence for daily vegetable intake across cities was 28.9%, ranging from 4.2% (Sultana, Peru) to 69.1% (Formosa, Argentina). The Supplementary Material (Figure S2 and Table S5) contains a full description of these prevalences. Prevalence variability across cities for fruit and vegetable daily intake prevalences was similar to the one described above for F&V (ICC_fruit_ = 0.65; 95% CI = 0.36-0.86; ICC_vegetable_ = 0.68; 95% CI = 0.42-0.86). Similar trends were observed according to contextual factors for both prevalences: 1) both prevalences were higher at the highest tertile of labour force participation and WA-Labor Force, 2) both prevalences were higher in cities with temperate weather and at lower GII, 3) for daily vegetable intake, prevalences were higher at higher city GDP. However, for daily intake of fruits, no significant pattern was observed for GDP, and a significant non-linear trend was observed for educational attainment and WA-Education (Table S6). We also performed an analysis excluding cities from Mexico, and the results were consistent with the main analysis (Table S6).

## Discussion

This is the first study to describe the variability of F&V intake at the city level across Latin American countries. Our data showed that the overall prevalence of daily F&V intake is relatively low; in one-third of the cities, less than one-half of the population consumed F&V daily. Within-country between-city heterogeneity accounted for approximately 33% of the variability in city-level F&V daily intake prevalence. Most of the variation in F&V prevalence was between countries. Generally, the prevalence was highest in Southern Cone cities from South America (Chile, Argentina, Southern Brazil), intermediate in Peru, Colombia, and Northern Brazil, and lowest in cities from Mexico and Central America. When stratifying the description by fruit and vegetable daily intake, Southern Cone cities had higher prevalences for daily intake of vegetables (and intermediate to high prevalences of fruit intake), whereas cities from Peru and Guatemala had higher prevalences for daily intake of fruits but low intake of vegetables (Supplementary Material, Figure S2). City-level F&V prevalences varied by city context, being higher in temperate (vs. arid and tropical) climates and cities with higher socio-economic development (labour force, city GDP) and women’s empowerment (WA-Labor force and GII). However, we did not find any linear pattern between city-level F&V and city educational level or city size (population and density).

The higher prevalence of F&V daily intake in cities with higher per capita GDP and labour force aligns with previous evidence at the country level^(6,7,28)^. However, no prior city-level studies exist to compare our results. GDP and labour force are closely related to service supply and demand, which could signal increased availability of F&V and consumer purchasing power. Also, the higher prevalence of F&V daily intake with WA-Labor force may be due to women having greater financial autonomy and more power in household decision-making, including healthier food choices^(17,29)^. Surprisingly, city education – which was included in the socio-economic domain and women’s empowerment (WA-Education) – did not show a pattern with city F&V daily intake. Education is an important determinant of overall health^(30)^; however, across varied Latin American contexts, educational advancements do not necessarily confer economic growth, development, and lower inequality^(31)^. Inequities in the quality of education might play a role. Even though educational attainment has increased in Latin American women, it has not been directly associated with higher labour force participation, job quality, or wages relative to men^(32,33)^. This gender gap in employment despite higher educational qualifications in women also has been recently reported in OECD countries^(34)^. Unlike compulsory education policies, higher female labour force participation, lower early marriage and lower gender inequality may also reflect structural changes in culture and gender roles. Therefore, measures of economic indexes and women’s autonomy might be stronger contextual determinants of F&V purchase on the population than education, but further research is warranted to explain these results.

Most of the literature shows higher odds of compliance with F&V intake in high-income countries, which is explained by a higher supply and economic access^(6,7,28)^. Similarly, the highest tertile of per capita GDP (≥17,300 GDP per capita; USD 2010) had the highest prevalence of F&V daily intake in our study. Although the median per capita GDP in our sample of cities was close to the world’s mean for 2011 (USD 13,580), it ranged from $959 to $116,572 (ppp) and had high variability within countries (IQR>$3,200)^(35)^. The latter heterogeneity reflects our region’s high economic inequity, with incomes below the sub-Saharan Africa region ($3,454) and higher than the European Union’s mean ($34,495), allowing us to detect patterns with F&V consumption. However, even in the highest GDP tertile, F&V daily intake prevalences had high variability. Also, this pattern was not statistically significant for fruit intake, suggesting that other factors are also important determinants (besides the city’s macroeconomic context). Other work has noted that Latin America has experienced exponential increases in the supply of ultra-processed foods (UPF), and thus the availability of UPF may account for some of this heterogeneity^(36,37)^.

We must highlight that almost all GII values observed in the cities included in our study were below the world’s mean (2015 GII: 0.48), especially when compared to sub-Saharan (0.57), South Asia (0.48), and Arab (0.52) regions^(38)^. However, unlike national estimates, we did not include rural areas or small urban areas (<100,000 habitants), which are expected to have higher GII and lower women’s empowerment. Therefore, our results do not meet all the criteria for comparability. Literature supports the hypothesis that women working out of home, high commuting times, and urbanisation increase the population demand for more convenient food (e.g. ultra-processed foods) at the expense of healthy foods such as F&V^(36,39)^. However, in our study, cities with higher women autonomy (WA-Labor force) and lower gender inequality showed higher prevalences of daily F&V intake. These results align with a previous review from 20 countries in sub-Saharan Africa, which also reported mainly positive effects of women’s empowerment on F&V intake^(40)^. Women tend to have a higher intake of F&V than men^(41,42)^, and higher empowerment may benefit household purchases and diet quality, although the time for preparing unprocessed foods is reduced. In Argentinean households, higher F&V were purchased by those in which women were the head of the household^(43)^. Also, previous evidence showed a lower prevalence of overweight and obesity in women living in Latin American cities with higher women’s empowerment, which is aligned with the pattern observed in this study^(16)^.

A higher prevalence of daily F&V intake in cities with a temperate climate zone compared to arid climates could be explained by more availability of F&V due to higher local production of F&V. However, we did not observe higher prevalences of daily intake in cities with tropical weather, which may also favour local production of F&V. Higher temperatures and humidity in tropical cities may accelerate the speed of fruit ripe and loss of organoleptic properties, reducing shelf-life and increasing subsequent waste during the supply chain and household storage compared to temperate weather^(44)^. In Latin America, approximately 50% of the original production of F&V is lost, with an important proportion occurring during the distribution and consumer stages^(44)^. Improving efficiency and conditions of the supply chain (post-harvest, transport, and retail) and access to suitable housing conditions (e.g. access to electricity, refrigerator, and piped water) may translate in higher intake by increasing shelf-life, guaranteeing safer consumption and reducing food waste from F&V and other perishable foods, especially in the urban environment and cities with disadvantaged climate conditions (e.g. arid zones and extreme temperatures)^(44,45)^. It is also essential to consider that F&V production, supply, and intake might also be affected by climate change^(19)^, which should be monitored through longitudinal studies in the future.

No previous description of F&V intake at the city level exists to compare our results. However, country-level data from the European Health Interview Survey reported a higher prevalence of daily intake of F&V in Europe compared to our study (median = 64% vs. 56%, respectively) with a high heterogeneity between countries (range = 26.2% to 82.6%)^(46)^. When stratifying the description, they reported considerably higher prevalences for daily fruit intake (median = 50% vs. 28%) and vegetables (median = 49% vs. 29%) compared to our data^(46)^. Previous evidence also supports that F&V intake, compared to Latin America, is higher in Western Europe^(47,48)^ and even in other low- and middle-income countries from other regions (Middle East & Central Asia, South & East Asia, and sub-Saharan Africa)^(48,49)^. Additionally, the country pattern observed in our study is similar to that found in the ELANS study through 24-h recalls. They reported the highest intake of F&V in Chile, the highest intake of fruits in Peru (followed by Chile and Argentina), and the highest intake of vegetables in Chile (followed by Argentina)^(5)^. This pattern might be explained by differences in culinary traditions and local or seasonal availability of F&V. Nevertheless, this pattern does not align with a previous study using the Global Dietary Database, which reported higher consumption of F&V in central and tropical Latin America^(48)^. However, they reported intake using another methodological approach, combining data from different surveys (grams per day) with FAO food balance sheets representing supply and considering legume intake within vegetables^(48)^.

The city-level summaries of F&V prevalences and patterns help identify inequalities across cities within and between countries. Policies that solely increase F&V production and supply (e.g. subsidies for producers) might not be enough if food waste is high and consumer demand is not increased locally^(45)^. Policies should also focus on subsidies to increase the consumers´ demand while strengthening the reach of local fresh foods (e.g. fresh produce markets, school meal programmes) and reducing F&V waste (e.g. through Food Banks, basic services supply), especially in low-income populations^(44,50,51)^. Evidence in the United States (‘Supplemental Nutrition Assistance Program’) and Brazil (‘Bolsa Familia’) reports a moderate positive impact of subsidies in F&V intake, especially when an economic incentive is given for buying them^(52–54)^. In Latin America, research shows higher F&V intake when residing in neighbourhoods with a high density of fresh produce markets or stores^(8,9,55)^.

### Strengths and limitations

This is one of the first large-scale comparative analyses of F&V intake at the city level. Further, we could describe intra-country variability and patterns of F&V compliance according to diverse city socio-economic and other contextual factors in diverse countries in the region. This is especially interesting for bigger countries and those with higher between-city variability in dietary intake, such as Brazil, Argentina, Chile, Perú, and Mexico.

This study has limitations. First, we used F&V frequency (daily intake) rather than other recommended measures to assess food intake, such as daily servings, because only survey data on F&V daily intake were plausible for standardisation across countries. Servings per day were not uniformly reported. Nevertheless, we estimate that results may be roughly similar if ‘guideline’ variables (such as a recommendation of ≥5 servings per day) had been available because the frequency of intake is strongly associated with an average intake of daily servings^(56)^. Moreover, due to the methodological differences and years of study, estimations of prevalences for daily intake of fruit and vegetables by country should be interpreted cautiously. However, comparability is still plausible within countries. This limitation highlights the need to standardise dietary data collection among health surveys in the region. Second, most of the data came from national health surveys aimed at making data representative at the national level rather than at the sub-national levels. For this reason, we decided to use a post-stratification method to compare the estimates between cities. Third, estimated prevalences for Guatemala may not represent an updated intake (the health survey was from 2002). However, it does not affect the observed patterns as it only represents one city. In the case of El Salvador, census data came from 2007. Nevertheless, we decided to keep both countries (Guatemala and El Salvador) to have some representation of cities (n = 4) from Central America. Finally, given the ecological design of the study, the results ignore within-city heterogeneity explained by neighbourhood and individual characteristics such as the socio-economic level.

### Conclusion

Prevalence of daily F&V intake varies across cities within Latin American countries and may be favoured by higher socio-economic development, lower gender inequity, and temperate weather. Interventions to improve fruit and vegetable intake in Latin America should be context-sensitive, considering the underlying local socio-economic, demographic, and biophysical inequalities.

## Supporting information

Valentino et al. supplementary material Valentino et al. supplementary material

## Data Availability

Survey data used in this paper are not publicly available; a link to the agency website can be accessed via https://drexel.edu/lac/data-evidence/data-acknowledgements/.

## Supplementary material

The supplementary material for this article can be found at https://doi.org/10.1017/jns.2024.93
