# Supplementary material for: Prevalence of daily fruit and vegetable intake by socio-economic characteristics, women’s empowerment, and climate zone: an ecological study in Latin American cities
Source: J Nutr Sci. 2025 Jan 15;14:e4. doi: 10.1017/jns.2024.93 (PMC11811847; doi:10.1017/jns.2024.93)
Supplement: Valentino et al. supplementary material [file S2048679024000934sup001.docx]

**Supplementary Material**

**Section 1 Methods2**

Section 1.1. Harmonization of dietary variables2

Section 1.2. Prevalence estimation: Indirect models and standardization2

Table S1. Original survey questions and harmonization4

Table S2. Health Surveys Design6

Table S3. Summary of data sources of contextual variables 8

Figure S1. Sample selection flowchart12

**Section 2 Results13**

Table S4. Characteristics of survey respondents 13

Figure S2. Prevalences of daily intake fruits and vegetable (stratified) 14

Table S5. Prevalences description for each city 15

Table S6. Sensitivity analyses 20

- 1. **Harmonization of fruit and vegetable intake**

Most countries directly asked the number of days per week of consumption (discrete variable ranging from 0 to 7), except for Colombia and Guatemala (Table S1). Guatemala asked for weekly and monthly consumption frequency, so, unlike the other countries, there are non-discrete data ranging from 0 to 7. In the case of Colombia, the frequency of consumption was asked with categories (twice a week, 5-6 times a week, etc.), so the average of the range included in the category was calculated. Mexico used a detailed Food Frequency Questionnaire that asked frequency for each item of fruit and vegetables, in which the most frequent item for each group was considered. Brazil and Colombia asked for cooked vegetables and salads separately, in which the one with highest frequency was considered for vegetables intake frequency. In the case of Peru, vegetables included only salads. Frequency of fruit and vegetable intake (days per week) was summed and topped to 7 days a week for building F&V intake.

- 1. **Indirect estimation of city prevalences of daily F&V consumption:**

**Predictive model:** We employed a mixed-effect logistic regression model to estimate the probability of complying with F&V consumption recommendation ($Y_{ij})$ where $i$ denotes an individual from city $j$.

$logit \left( Y_{ij} \right)=\beta_{0;ij}+\beta_{1}A_{ij} +\beta_{2}E_{ij}+\beta_{3}S_{ij}$ (1)

We use random intercepts for the cities such that $\beta_{0;ij}=\gamma_{00} +v_{0j},$ where $\gamma_{00}$ corresponds to the overall (grand) intercept and $v_{0j}$ corresponds to city-specific random deviations from the grand intercept, assumed to be normally distributed with mean 0 and variance $\tau^{2}$. Our model includes auxiliary adjustment variables: sex (2 groups), age (categorical variable with 5 groups: 20-29; 30-39; 40-49; 50- 59; 60-69), and educational level (4 groups). The regression coefficients $\beta_{1}$, $\beta_{2}, and \beta_{3}$ respectively denote the effects of age$(A)$, education$(E)$, and sex ($S)$. The models were stratified by country.

Using the estimated model parameters, we obtained the mean predicted probability of complying with the F&V consumption recommendation ($\hat{Y_{asj}})$ for each age group $a$, sex $s$, and city $j$. Since the random intercepts for all cities (in a given country) shared the common variance, it helped to reduce the variance and obtain stable estimates.

**Post-stratification:** To allow estimates to be comparable across cities and countries, we standardized our estimates of the probability of complying with daily F&V consumption using the age and sex distribution of the population in all SALURBAL cities combined. The standardization/post-stratification weights were calculated based on the 2010 census population projections in all SALURBAL cities. Specifically, using the estimated age-, sex-, and city- specific estimates $\hat{Y_{asj}}$, we obtained the city-specific estimates of the proportion of the population that meets the F&V consumption recommendation $(\hat{\theta}_{j})$ as the weighted sum:

$\hat{\theta}_{j}= \sum_{as} \hat{Y_{asj}}\times W_{as},$ (2)

where, $W_{as}$ is the post-stratification weight in age and sex stratum representing proportion of the population in that age-sex group.

**Table S1. Summary of the harmonization process of fruit and vegetable data from original health surveys**

|  | **Original Health Survey Questionnaire** | | **Harmonization process** | | |
| --- | --- | --- | --- | --- | --- |
|  | **Fruit intake frequency** | **Vegetable intake frequency** | **Fruit intake frequency** | **Vegetable intake frequency** | **F&V intake frequency** |
| **Argentina** | In a typical week, how many days do you eat fruits? Response: Number of days (Numeric), Never, Doesn't know | In a typical week, how many days do you eat vegetables? Response: Number of days (Numeric), Never, Doesn't know | 0-7 days | 0-7 days | 1) Sum of Fruit intake frequency and Vegetable intake frequency capped to 7 days (Numeric: 0-7 days)  2)F&V daily intake (binary):  Yes: F&V=7 days  No:  F&V<7 days |
| **Brazil** | How many days a week do you eat fruits?  Response: number of days | How many days of the Q1. How many days of the week do you eat lettuce and tomato salad or any other vegetable or raw; Q2. On how many days of the week do you usually eat cooked vegetables or vegetables such as cabbage, carrots, chayote, eggplant, zucchini? (not counting potatoes, cassava, or yams) vegetable salad? Response: number of days | 0-7 days | 0-7 days of most frequent vegetable (raw or cooked) |  |
| **Chile** | In a typical week, how may days do you eat fruits? Response: number of days | In a typical week, how many days do you eat vegetables or vegetable salads? Don’t include potatoes or legumes. Response: number of days | 0-7 days | 0-7 days |  |
| **Colombia** | How often do you eat these food ítems? [whole fruits]. Responses: Less than once a month, once a month, 2-3 times per month, once a week, twice a week, three to four times a week, five to six times week, once a day, twice a day three times or more per day | Q1. How often do you eat these food ítems? [Cooked vegetables]. Q2. How often do you eat these food ítems?[Raw vegetables]. Responses (Q1 and Q2): Less than once a month, once a month, 2-3 times per month, once a week, twice a week, three to four times a week, five to six times week, once a day, twice a day three times or more per day | 0-7 days (ranges were averaged: 0, 0.5, 1, 2, 3.5, 5.5, 7) | 0-7 days of most frequent vegetable (raw or cooked) (ranges were averaged: 0, 0.5, 1,2,3.5,5.5, 7) |  |
| **El Salvador** | In a typical week, how many days do you eat fruits? Response: number of days, doesn’t know | In a typical week, how many days do you eat vegetables or vegetable salads? Response: number of days, doesn’t know | 0-7 days | 0-7 days |  |
| **Guatemala** | In the last month, have you eaten Fruits without counting juices? How much? (Frequency). Responses: Never, daily, once per week, twice per week, 3 times per week, 4 times per week, 5 times per week, 6 times per week, 1 time per month, 2 times per month, 3 times per month, Do not know | In the last month, have you eaten vegetables, herbs, vegetables? How much? (Frequency). Responses: Never, daily, once per week, twice per week, 3 times per week, 4 times per week, 5 times per week, 6 times per week, 1 time per month, 2 times per month, 3 times per month, Do not know | 0-7 days (monthly frequency divided by 4: 0, 0.25, 0.5, 0.75, 1, 2, 3, 4,5,6, 7) | 0-7 days (monthly frequency divided by 4: 0, 0.25, 0.5, 0.75, 1, 2, 3, 4,5,6, 7) |  |
| **Mexico** | Full FFQ: In the last seven days.....How many days did you eat (specific fruit)? (included 13 fresh fruit items) Response: 0 to 7 days | Full FFQ: In the last seven days.....How many days did you eat (specific vegetable)? (included 17 vegetable items). Response: 0 to 7 days | 0-7 days of most frequent fruit | 0-7 days of most frequent vegetable: |  |
| **Peru** | In the past 7 days, or from last [date] until yesterday, how many days did you eat fruits? If you have eaten fruits. Response: number of days, did not eat, doesn't know | In the past 7 days, or from last [date] until yesterday, how many days did you eat vegetables? If you have eaten fruits. Response: number of days, did not eat, doesn't know | 0-7 days | 0-7 days |  |

F&V, Fruit and vegetable; FFQ, Food Frequency Questionnaire

**Table S2. Description of health surveys including data availability, sample design, representativity and SALURBAL cities included**

| **Country** | | | | | | | | |
| --- | --- | --- | --- | --- | --- | --- | --- | --- |
|  | **Argentina** | **Brazil** | **Chile** | **Colombia** | **El Salvador** | **Guatemala** | **Mexico** | **Peru** |
| **National Health Survey name and year** | Encuesta Nacional de Factores de  Riesgo, ENFR 2013 | Pesquisa  Nacional de Saúde, PNS  2013 | Encuesta Nacional de Salud  2017 | Encuesta Nacional de Salud 2015 | ENECA 2014-2015 | CAMDI  2002 | ENSANUT  2018 | ENDESA  2016 |
| **Sample characteristics of the survey** | Age ≥ 18 y Total N: 32.365  N in SALURBAL:  **21,451** | Age: all  Total N: 64.308 adults ≥18 years  N in SALURBAL:  40.703  N with lifestyle module (P): **29,353** | Age: ≥15 years  Total N: 6.233  N in SALURBAL:  3.805 N with diet module: **4,899** | Age: 0 - 69 years  Total N: 87.819  18 - 69 years  N in SALURBAL: **36,593**  18 - 69 años | Age: ≥20 years  Total N: 4.817  N in SALURBAL:  **1.546** | Age: ≥20 years  Total N: 1.397  N in SALURBAL:  **1,397** | Age: all  Total N: 46.277  ≥ 18 years  N in SALURBAL:  27,118 ≥ 18 years  N with diet module: **10,095** | Age: all  Total N: 122.368 (adults ≥18 years N=32.158)  N in SALURBAL:  **11,929** |
| **Representa-tivity** | National, 6  regions, 23 provinces,  Ciudad Autonoma de Buenos Aires, and metropolitan areas of >500,000 inhabitants | Regions (5) States or federation units (27), state capitals (27), urban and rural, metropolitan  areas and development  integrated areas | National, Regions (15), urban/rural | National, Regions (15),  urban/rural | National, Urban Rural | Villa Nueva Municipio | National, state, metropolitan areas, urban/rural,  high/low SES | National, Urban National, Rural National, Natural Region: Lima Metropolitan area, coast/mountain/jungle |
| **Sample design** | Multistage [Aglomerado censal; area groups of radio censales); household; person 18 years or older]  Stratified [population  size; education level of  head of household] | Multistage [census tracts  or groups of census tracts; households; person 18 years or older]  Stratified [capital city, metropolitan region, or  integrated economic development region, then  rest of municipalities; Urban/rural; total household  income] | Multistage [Comunas; Segments within comunas; household; person 15 years or older]  Stratified [urban/rural  with three groups of population sizes] | Multistage [Municipalities  or combination of municipalities if small; Rural  blocks or sections;  household segments;  person adults 18-69 and children 17 and under]  Stratified [region; urbanization of municipal seats; urban/rural municipal  population; unsatisfied  basic needs] | Two-stage [Segmento  censal, groups of dwellings (compacto); all  household members 20  years and older] | Multistage [Segmento  censal, groups of dwellings (compacto); all  household members 20  years and older] | Multistage [AGEB; Manzana (urban) or pseudo-manzanas within localidades (rural); Households; 1 person within each of the groups (0-4 years, 5-9 years, 10-19 years, 20 years and older, recent medical service user)]  Stratified [SES of AGEB at the state level] | Multistage [Conglomerado (set of census blocks – urban) ; households; One person within each of the groups (>15 years, females 15-49 years, children <5  years, children <12 years)] Stratified [Department;  Urban/Rural] |
| **N of cities** | 33 | 27 | 21 | 35 | 3 | 1 | 91 | 23 |
| **Sample per city, median (p25-p75)** | 783  (379-1214) | 927 (834-1179) | 133 (72-237) | 331  (114-597) | 352 (164-1030) | 1397 (NA) | 190 (89-388) | 373 (168-660) |

AGEB, Geo-Statistic Basic Area; SES, Socioeconomic status; ENECA, Encuesta Nacional de Enfermedades Crónicas no transmisibles en Población Adulta de El Salvador; CAMDI, Encuesta Multinacional de Diabetes y Factores de Riesgo; ENSANUT, Encuesta Nacional de Salud y Nutrición; ENDESA, Encuesta Nacional de Demografia y Salud. Adapted from SALURBAL Data Core documents. Bold letter indicate sample with F&V data.

**Table S3. Data sources and interpretation for dietary and sociodemographic variables**

| **Role** | **Level** | **Variable** | **Data Source** | **Definition and interpretation** | **Relevant details** | **Year of data collection** |
| --- | --- | --- | --- | --- | --- | --- |
| **Exposure: Socioeconomic factors** | **City-level** | **Educational attainment** | Census. Highest grade or educational level completed. | Proportion of population ≥25 years old who completed secondary education | IPUMS criterio for harmonization. | Closest census year:  Argentina (2010), Brazil (2010), Chile (2017), Colombia (2018), El Salvador (2007),  Guatemala (2002), Mexico (2020), Peru (2017) |
|  |  | **Labor force** | Census. Employment status during the last week (worked at least one hour or was looking for a job). | Proportion of population ≥15 years old who is working or actively seeking for a job | IPUMS criteria and harmonized to population ≥15 years old |  |
|  |  | **GDP per capita** | Subnational estimations from previous studies (Genniaoli et al., 2013; Kummu et al., 2018) | Constant 2011 international USD (purchasing power parity) a year per inhabitant | Some cities from the same region share the same GDP estimation. Our dataset had 116 unique GDP values for 144 cities (16 GDP values were shared by 2 cities, 4 values by 3 cities, and 1 value by 5 cities). | We matched the year GDP per capita to the year of the Health Survey for each country, and used 2015 for those with Health Surveys posterior to 2015 (Chile, Mexico, Peru) |
| **Exposure: Women Empowerment indexes** | **City-level** | **WA-Labor force** | Census Variables. The scores reflect factors identified using factor analysis with L1AD data. These domains include women’s labor force participation, women’s education, marriage among girls aged 15 to 17. Early marriage was reverse reversed coded so higher scores signify higher achievements. Z-scores were created of each indicator included in the two domains and summed them together to derive a score for each factor. (Braverman-Bronstein et al., 2023) | Includes proportion of women aged 15 and older in the labor force and the proportion of women between 15 and 17 years old who are married. Higher score indicate higher achievements in women autonomy. |  | Censuses: Argentina (2010), Brazil (2010), Chile (2017), Colombia (2018), El Salvador (2007), Guatemala (2018), Mexico (2010), Peru (2017) |
|  |  | **WA-Education** |  | Includes porportion of women aged 25 and older with at least high school education and proportion of women aged 25 and older with at least university education. Higher scores indicate higher achievements in education. |  |  |
|  |  | **Gender Inequality Index** | Census and Electoral Tribunals data. This measure was adapted from the Gender Inequality Index developed by the United Nations Development Fund (UNDP), which uses country-level data. We used two of the three domains: empowerment (education and government: % female and male population 25+ with at least high school education, and the % women and men in mayoral positions in the government for the period of 2010-2015), and the labor market (% female and male labor force participation). (Braverman-Bronstein et al., 2023) | This index is a measure of the loss in potential human development due to inequality between men and women. The index ranges from 0, indicating a context where women and men fare equally, to 1, indicating a context in which one gender fares poorly compared to the other in all measured dimensions. (more details: Gender Inequality Index (GII) \| Human Development Reports: <http://hdr.undp.org/en/content/gender-inequality-index-gii>.) | The third domain of health (adolescent birth rate and maternal mortality) was not available for Peru. Results were similar when using the three-domain GII at the expense of excluding Peru cities | Same census years as WA scores + Political Participation: Argentina (2015, 2017), Brazil (2012), Chile (2012), Colombia (2011), El Salvador (2012), Guatemala (2011), Mexico (2010-2014), Peru (2015) |
| **Exposure: Demographic characteristics and climate zone** | **City-level** | **City size (total population)** | Census. Intercensal total population projections (Quistberg et al., 2019). | Total population in each city. |  | We matched the year of the population projection to the same year of the Health Survey for each country. |
|  |  | **Population density** | Census (total population) and city built-up area based on the precise built-up urban extent identified systematically using satellite imager (Quistberg et al., 2019). | Population per square kilometer in all urban patches or administrative area inside the geographic boundary of the city. |  |  |
|  |  | **Climate zone** | Assigned by Köppen system, which considers four main categories (Kottek et al, 2006; Rubel & Kottek 2010: <http://koeppen-geiger.vu-wien.ac.at/shifts.htm> ) | Major climate zone of the city indicating seasonal precipitations and temperature patterns: tropical, arid, temperate, and polar. |  |  |
| **Outcome** | **City-level** | **Prevalence of daily intake of F&V** | Health Surveys. Individual-level binary variable "Days per week with fruit and vegetable consumption = 7 days" built by summing days per week with fruit consumption and days per week with vegetable consumption (both available in health surveys) and capped at 7 days. Then, prevalence was estimated using the binary variable through indirect models and standardized by age and sex. | Proportion of the population with fruit and vegetable intake 7 days per week standardized by age and sex. | Mexico used a complete FFQ: frequency of the most frequent fruit and most frequent vegetable were considered to avoid overestimation. Peru only considered raw vegetable consumption. | Argentina (2013), Brazil (2013), Chile (2017), Colombia (2015), El Salvador (2014),  Guatemala (2002), Mexico (2018), Peru (2016) |

IPUMS, Integrated Public Use Microdata Series; FFQ, Food Frequency Questionnaire.

**Figure S1. Sample Selection Flowchart**

**
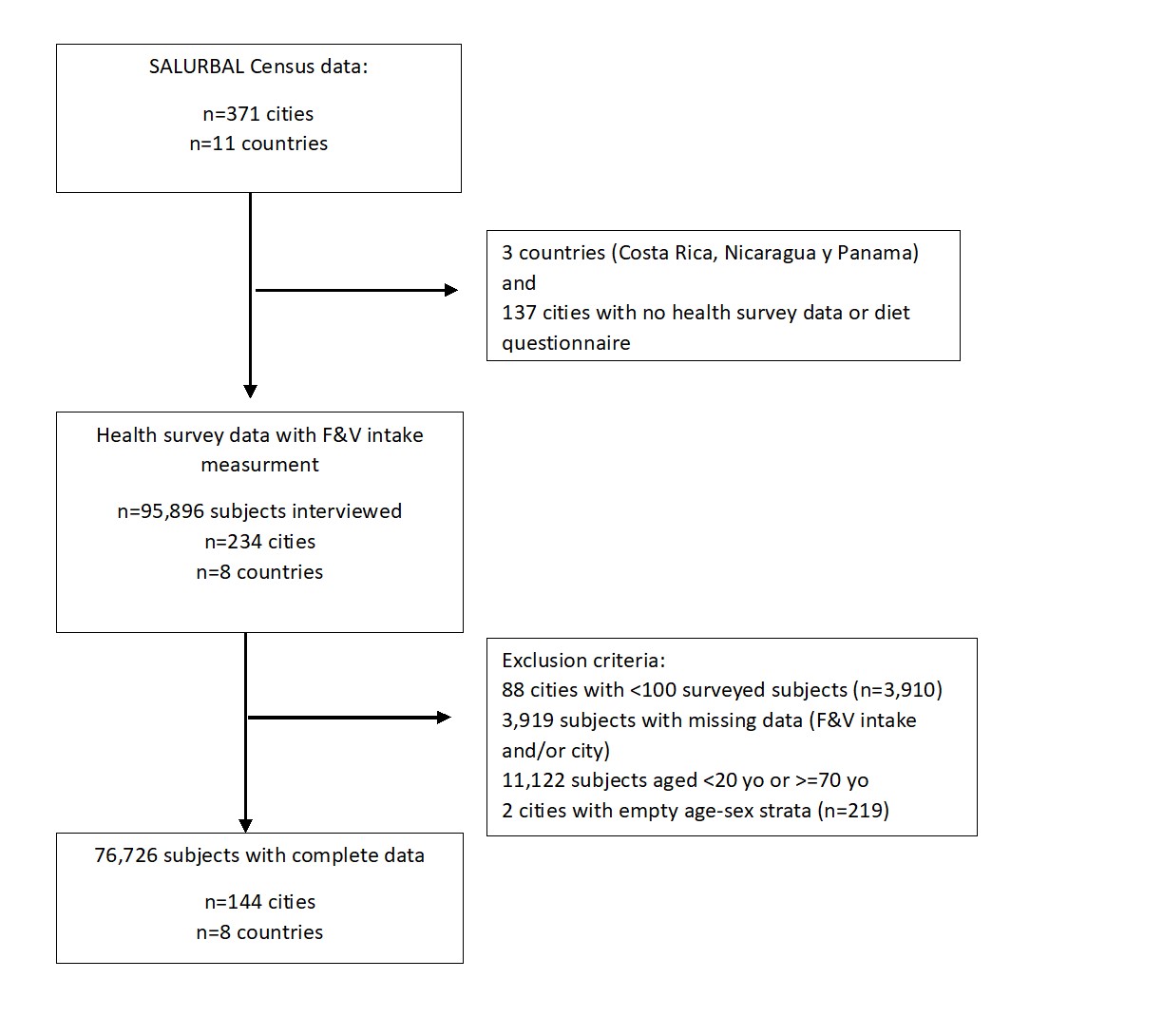
**

**Table S4. Description of the survey participants**

|  | All countries | Argentina | Brazil | Chile | Colombia | Guatemala | Mexico | Peru | El Salvador |
| --- | --- | --- | --- | --- | --- | --- | --- | --- | --- |
| **Survey data** | | | | | | | | | |
| N°of participants | 76,726 | 17,986 | 33,282 | 2,565 | 3,297 | 1,308 | 6,236 | 10,675 | 1,377 |
| N° of participants by city, Median (IQR) | 319  (527) | 454  (816) | 967  (816) | 170  (132) | 176  (146) | 1,308 | 150  (125) | 318  (356) | 313  (768) |
| Age, years old | 40.8  (13.4) | 41.4  (13.8) | 41.1  (13.1) | 45.1  (14.5) | 38.5  (12.8) | 37.7  (13.1) | 42.2  (13.6) | 38.2  (12.6) | 41.9  (13.8) |
| Female, n (%) | 44,335  (58%) | 9,942 (55%) | 19,263 (58%) | 1,606 (59%) | 1,954  (59%) | 887 (68%) | 3,535 (57%) | 6,209 (58%) | 939 (68%) |
| High school education, n (%) | 41,785 (55%) | 10,373 (58%) | 18,355 (55%) | 1,270 (50%) | 1,080 (33%) | 247 (19%) | 2,395 (38%) | 7,665 (72%) | 400 (30%) |
| Daily F&V intake, n (%) | 48,913 (64%) | 12,172 (68%) | 23,173 (70%) | 2,005 (78%) | 1,675 (51%) | 836 (64%) | 2,630 (42%) | 5,741 (54%) | 681 (50%) |
| Daily intake of fruits, n (%) | 24,119 (31%) | 5,930 (33%) | 10,312 (31%) | 940 (37%) | 722 (22%) | 609 (47%) | 1,187 (19%) | 4,033 (38%) | 386 (28%) |
| Daily intake of vegetables, n (%) | 25,187 (33%) | 6,914 (38%) | 12,484 (38%) | 1,430 (56%) | 1,006 (31%) | 413 (32%) | 1,146 (18%) | 1,611 (15%) | 183 (13%) |

Data expressed as mean and standard deviation for continuous variables and absolute and relative frequency for categorical variables. Number of participants by city is expressed as median and interquartile range (IQR).

**Figure S2. Prevalences of daily intake of fruit and vegetable for 144 Latin-American cities**


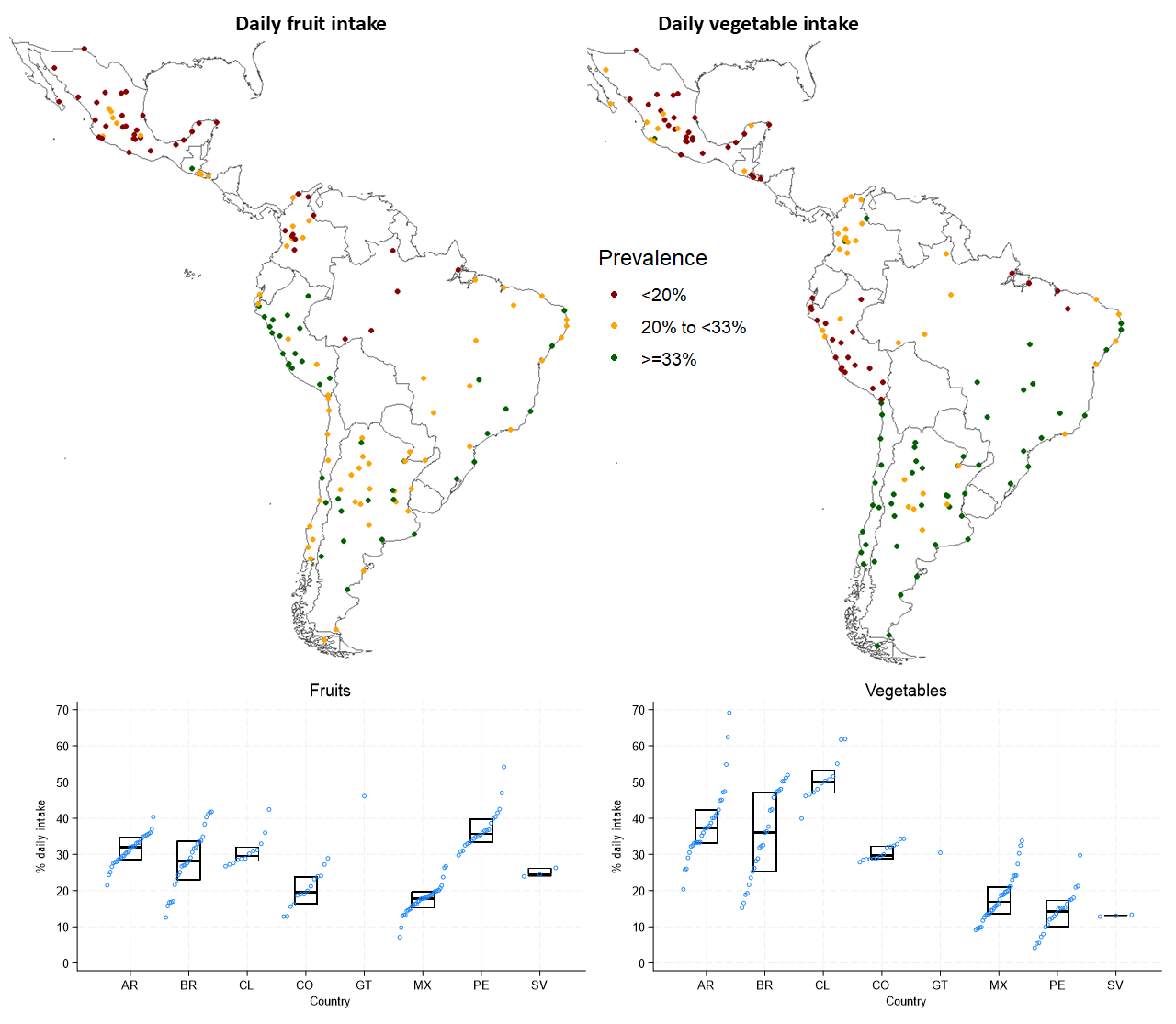


AR, Argentina; BR, Brazil; CL, Chile; CO, Colombia; GT, Guatemala; MX, Mexico; PE, Peru; SV, El Salvador. Daily F&V intake was derived from the following years of national health surveys: 2013 for Argentina and Brazil, 2002 for Guatemala, 2015 for Colombia and El Salvador, 2016 for Peru, and 2018 for Mexico. On the graphs, each blue dots indicates a city.

**Table S5. Prevalences of daily fruit and vegetable intake by city and country (population aged 20 to 69 years old)**

|  |  | **Sample** | | **% Daily F&V intake** | | **% Daily fruit intake** | **% Daily vegetable intake** |
| --- | --- | --- | --- | --- | --- | --- | --- |
| **Country** | **City Name** | **N** | **N** | **Indirect estimation standardized by sex and age (95% CI)** | **Direct estimation from survey**  **(95% CI)** | **Indirect estimation standardized by sex and age (95% CI)** | **Indirect estimation standardized by sex and age (95% CI)** |
|  |  | **(total)** | **(20-69 y)** |  |  |  |  |
| Argentina | Bahia Blanca | 417 | 355 | 70.0 (69.2; 70.7) | 72.9 (68.5; 79.6) | 40.4 (39.3; 41.5) | 40.7 (39.9; 41.5) |
| Argentina | Mar del Plata | 722 | 575 | 63.1 (62.5; 63.7) | 68.3 (64.8; 71.7) | 36.0 (35.2; 36.8) | 33.4 (32.8; 34.0) |
| Argentina | San Nicolas de los Arroyos | 362 | 294 | 65.5 (64.8; 66.3) | 70.4 (65.7; 75.2) | 32.1 (31.1; 33.1) | 37.2 (36.4; 38.0) |
| Argentina | Catamarca | 408 | 357 | 58.4 (57.7; 59.1) | 60.3 (55.5; 65.1) | 24.3 (23.5; 25.1) | 35.2 (34.5; 36.0) |
| Argentina | Cordoba | 923 | 782 | 58.4 (58.0; 58.9) | 62.8 (59.7; 66.0) | 29.6 (29.0; 30.2) | 29.1 (28.6; 29.5) |
| Argentina | Rio Cuarto | 379 | 304 | 69.1 (68.3; 69.8) | 73.1 (68.5; 77.7) | 35.7 (34.7; 36.7) | 37.6 (36.8; 38.4) |
| Argentina | Resistencia | 368 | 322 | 72.0 (71.4; 72.7) | 74.2 (69.6; 78.7) | 33.3 (32.2; 34.3) | 44.9 (44.0; 45.8) |
| Argentina | Comodoro Rivadavia | 477 | 423 | 72.3 (71.8; 72.9) | 74.6 (70.7; 78.6) | 33.1 (32.3; 33.9) | 41.3 (40.6; 42.0) |
| Argentina | Corrientes | 665 | 557 | 60.0 (59.4; 60.6) | 62.9 (59.1; 66.6) | 25.1 (24.5; 25.8) | 30.5 (29.9; 31.1) |
| Argentina | Buenos Aires | 3870 | 3227 | 64.1 (63.8; 64.3) | 67.8 (66.3; 69.3) | 32.3 (32.0; 32.6) | 33.3 (33.1; 33.5) |
| Argentina | Concordia | 417 | 358 | 70.9 (70.3; 71.5) | 73.6 (69.3; 77.9) | 32.0 (31.1; 32.9) | 47.1 (46.4; 47.9) |
| Argentina | Parana | 509 | 436 | 68.4 (67.9; 69.0) | 70.9 (66.9; 74.9) | 28.5 (27.7; 29.3) | 40.1 (39.4; 40.8) |
| Argentina | Formosa | 528 | 454 | 85.8 (85.5; 86.1) | 87.7 (84.8; 90.6) | 30.5 (29.8; 31.3) | 69.1 (68.5; 69.7) |
| Argentina | Jujuy | 495 | 432 | 76.1 (75.7; 76.6) | 78.2 (74.5; 81.9) | 29.7 (28.9; 30.4) | 54.8 (54.1; 55.6) |
| Argentina | Santa Rosa | 547 | 462 | 62.9 (62.3; 63.4) | 65.4 (61.4; 69.5) | 30.3 (29.5; 31.1) | 20.4 (19.9; 20.8) |
| Argentina | La Rioja | 531 | 466 | 55.2 (54.6; 55.8) | 53.9 (49.5; 58.2) | 21.5 (20.9; 22.1) | 25.7 (25.2; 26.2) |
| Argentina | Mendoza | 888 | 760 | 69.5 (69.0; 70.0) | 72.7 (69.8; 75.7) | 35.4 (34.7; 36.1) | 45.1 (44.5; 45.6) |
| Argentina | San Rafael | 158 | 127 | 67.4 (66.2; 68.6) | 74.1 (67.0; 81.1) | 34.8 (33.1; 36.4) | 38.7 (37.4; 40.1) |
| Argentina | Posadas | 418 | 372 | 82.7 (82.2; 83.1) | 84.9 (81.4; 88.4) | 27.6 (26.8; 28.5) | 62.4 (61.7; 63.1) |
| Argentina | Neuquen-Plottier-Cipolletti | 1211 | 1015 | 66.8 (66.4; 67.1) | 69.0 (66.4; 71.7) | 35.2 (34.6; 35.7) | 37.3 (36.8; 37.7) |
| Argentina | San Carlos de Bariloche | 480 | 407 | 71.2 (70.5; 71.8) | 72.9 (68.9; 77.0) | 35.0 (34.0; 35.9) | 42.3 (41.6; 43.1) |
| Argentina | Salta | 709 | 609 | 74.2 (73.8; 74.7) | 76.0 (72.8; 79.3) | 34.1 (33.4; 34.9) | 47.4 (46.8; 48.0) |
| Argentina | Santiago del Estero | 691 | 596 | 64.0 (63.5; 64.5) | 66.3 (62.7; 69.9) | 26.6 (25.9; 27.3) | 37.9 (37.3; 38.5) |
| Argentina | Rosario | 948 | 781 | 65.9 (65.4; 66.3) | 67.9 (64.9; 71.0) | 37.0 (36.3; 37.7) | 32.2 (31.7; 32.7) |
| Argentina | Santa Fe | 549 | 474 | 68.9 (68.4; 69.5) | 70.9 (67.0; 74.7) | 33.2 (32.4; 34.0) | 40.1 (39.5; 40.8) |
| Argentina | San Juan | 648 | 527 | 67.6 (67.1; 68.1) | 69.1 (65.5; 72.8) | 30.8 (30.1; 31.6) | 33.3 (32.7; 33.9) |
| Argentina | San Luis | 492 | 431 | 63.3 (62.7; 63.9) | 63.6 (59.3; 68.0) | 29.1 (28.3; 29.9) | 32.4 (31.8; 33.1) |
| Argentina | Villa Mercedes | 263 | 229 | 60.8 (60.0; 61.5) | 63.5 (57.6; 69.4) | 27.9 (26.8; 28.9) | 26.0 (25.3; 26.8) |
| Argentina | San Miguel de Tucuman-Tafi Viejo | 1082 | 935 | 66.8 (66.4; 67.1) | 68.9 (66.0; 71.7) | 27.9 (27.4; 28.4) | 36.0 (35.5; 36.4) |
| Argentina | Rawson-Trelew | 490 | 433 | 63.3 (62.7; 63.9) | 65.5 (61.2; 69.8) | 32.2 (31.4; 33.1) | 33.3 (32.6; 33.9) |
| Argentina | Rio Gallegos | 550 | 486 | 63.7 (63.1; 64.2) | 65.5 (61.4; 69.5) | 28.7 (27.9; 29.4) | 33.1 (32.5; 33.7) |
| Brazil | Rio Branco | 919 | 832 | 51.1 (50.5; 51.7) | 51.9 (48.6; 55.2) | 16.8 (16.4; 17.3) | 26.2 (25.8; 26.6) |
| Brazil | Maceio | 1006 | 896 | 62.5 (61.9; 63.0) | 64.7 (61.7; 67.7) | 24.3 (23.7; 24.9) | 31.9 (31.4; 32.4) |
| Brazil | Macapa | 1009 | 878 | 43.5 (43.0; 44.1) | 43.3 (40.1; 46.5) | 12.6 (12.3; 13.0) | 19.3 (19.0; 19.6) |
| Brazil | Manaus | 1761 | 1588 | 57.5 (57.1; 57.9) | 55.9 (53.6; 58.3) | 17.0 (16.7; 17.3) | 21.6 (21.3; 21.9) |
| Brazil | Salvador | 1759 | 1590 | 64.1 (63.7; 64.5) | 65.6 (63.3; 67.9) | 27.1 (26.7; 27.6) | 28.9 (28.5; 29.2) |
| Brazil | Fortaleza | 1642 | 1425 | 58.6 (58.2; 59.0) | 60.5 (58.1; 63.0) | 31.7 (31.2; 32.2) | 23.5 (23.2; 23.8) |
| Brazil | Brasilia | 1811 | 1633 | 77.9 (77.6; 78.2) | 78.7 (76.8; 80.6) | 41.6 (41.1; 42.2) | 48.0 (47.6; 48.4) |
| Brazil | Vitoria | 754 | 655 | 81.6 (81.2; 82.1) | 84.1 (81.4; 86.7) | 41.8 (40.9; 42.7) | 51.2 (50.5; 51.8) |
| Brazil | Goiania | 1493 | 1348 | 78.3 (78.0; 78.6) | 78.4 (76.3; 80.6) | 27.5 (27.0; 28.0) | 50.2 (49.7; 50.6) |
| Brazil | Sao Luis | 1083 | 967 | 63.8 (63.3; 64.3) | 64.8 (61.9; 67.7) | 26.7 (26.1; 27.3) | 16.6 (16.3; 16.9) |
| Brazil | Cuiaba | 763 | 698 | 76.3 (75.8; 76.8) | 76.4 (73.3; 79.5) | 25.1 (24.4; 25.8) | 46.6 (46.0; 47.2) |
| Brazil | Campo Grande | 865 | 774 | 71.2 (70.7; 71.6) | 72.1 (69.1; 75.2) | 27.0 (26.4; 27.6) | 50.3 (49.7; 50.8) |
| Brazil | Belo Horizonte | 2729 | 2385 | 80.8 (80.5; 81.0) | 82.4 (81.0; 83.9) | 33.8 (33.3; 34.2) | 51.9 (51.6; 52.2) |
| Brazil | Belem | 1178 | 1058 | 45.5 (45.0; 45.9) | 46.3 (43.3; 49.2) | 22.9 (22.4; 23.4) | 15.3 (15.0; 15.5) |
| Brazil | Joao Pessoa | 1212 | 1071 | 67.3 (66.8; 67.8) | 68.3 (65.6; 71.0) | 30.6 (30.0; 31.2) | 37.7 (37.2; 38.2) |
| Brazil | Curitiba | 2097 | 1859 | 78.3 (78.0; 78.6) | 78.8 (77.0; 80.6) | 33.0 (32.5; 33.4) | 47.3 (47.0; 47.7) |
| Brazil | Recife | 1542 | 1359 | 68.4 (68.0; 68.9) | 69.8 (67.5; 72.2) | 28.2 (27.7; 28.8) | 36.3 (35.9; 36.8) |
| Brazil | Teresina | 987 | 879 | 56.3 (55.8; 56.9) | 57.5 (54.4; 60.7) | 21.6 (21.1; 22.1) | 18.9 (18.5; 19.2) |
| Brazil | Rio de Janeiro | 2609 | 2224 | 71.0 (70.7; 71.3) | 74.0 (72.3; 75.7) | 29.1 (28.7; 29.5) | 32.3 (32.0; 32.6) |
| Brazil | Natal | 926 | 825 | 67.2 (66.7; 67.7) | 68.5 (65.4; 71.5) | 34.9 (34.2; 35.6) | 32.6 (32.1; 33.0) |
| Brazil | Porto Alegre | 1962 | 1681 | 78.7 (78.4; 79.0) | 80.6 (78.9; 82.4) | 40.3 (39.7; 40.8) | 47.7 (47.3; 48.1) |
| Brazil | Porto Velho | 837 | 763 | 53.1 (52.6; 53.7) | 53.9 (50.4; 57.3) | 16.8 (16.3; 17.2) | 25.2 (24.8; 25.6) |
| Brazil | Boa Vista | 957 | 868 | 56.1 (55.5; 56.6) | 55.9 (52.7; 59.1) | 15.8 (15.4; 16.1) | 28.2 (27.8; 28.6) |
| Brazil | Florianopolis | 861 | 758 | 75.6 (75.1; 76.1) | 76.9 (74.0; 79.8) | 41.1 (40.3; 42.0) | 45.7 (45.1; 46.3) |
| Brazil | Sao Paulo | 3521 | 3060 | 75.4 (75.1; 75.6) | 77.6 (76.2; 79.0) | 38.4 (38.0; 38.7) | 42.4 (42.1; 42.7) |
| Brazil | Aracaju | 676 | 607 | 72.0 (71.4; 72.6) | 73.7 (70.3; 77.0) | 33.5 (32.7; 34.4) | 36.0 (35.4; 36.6) |
| Brazil | Palmas | 662 | 601 | 78.4 (77.9; 78.9) | 77.8 (74.5; 81.0) | 31.9 (31.1; 32.7) | 42.2 (41.6; 42.8) |
| Chile | Arica | 346 | 286 | 79.1 (78.6; 79.6) | 83.8 (79.8; 87.8) | 28.8 (27.8; 29.8) | 55.0 (54.0; 56.1) |
| Chile | Iquique | 288 | 246 | 72.7 (72.0; 73.3) | 74.3 (69.2; 79.4) | 28.5 (27.4; 29.6) | 48.0 (46.9; 49.2) |
| Chile | Antofagasta | 192 | 155 | 71.7 (71.0; 72.3) | 72.9 (66.5; 79.3) | 31.2 (29.9; 32.6) | 47.1 (45.7; 48.5) |
| Chile | Copiapo | 165 | 141 | 75.5 (74.7; 76.3) | 78.8 (72.4; 85.2) | 26.7 (25.4; 28.0) | 51.5 (50.1; 53.0) |
| Chile | La Serena-Coquimbo | 173 | 131 | 67.9 (66.8; 69.0) | 71.1 (64.1; 78.1) | 36.0 (34.1; 37.9) | 49.6 (47.8; 51.5) |
| Chile | Valparaiso-Viña del Mar | 330 | 252 | 81.1 (80.7; 81.6) | 87.6 (83.9; 91.2) | 31.0 (29.9; 32.1) | 61.8 (60.8; 62.9) |
| Chile | Santiago | 751 | 602 | 79.9 (79.6; 80.3) | 84.4 (81.8; 87.1) | 42.4 (41.6; 43.2) | 61.7 (61.0; 62.4) |
| Chile | Talca | 109 | 86 |  | 79.8 (72.1; 87.5) |  |  |
| Chile | Concepcion | 319 | 259 | 71.8 (71.2; 72.4) | 74.0 (69.1; 78.9) | 33.0 (31.9; 34.1) | 50.3 (49.2; 51.4) |
| Chile | Temuco | 133 | 105 | 75.9 (75.1; 76.7) | 81.2 (74.3; 88.1) | 30.2 (28.5; 31.9) | 50.7 (49.0; 52.4) |
| Chile | Valdivia | 140 | 117 | 72.0 (71.0; 73.0) | 74.3 (66.8; 81.7) | 27.6 (26.1; 29.2) | 46.6 (44.7; 48.4) |
| Chile | Puerto Montt | 108 | 87 | 73.2 (72.3; 74.1) | 75.9 (67.7; 84.2) | 27.2 (25.6; 28.9) | 39.9 (38.1; 41.8) |
| Chile | Punta Arenas | 237 | 184 | 70.0 (69.2; 70.8) | 73.0 (67.3; 78.7) | 28.9 (27.5; 30.2) | 46.1 (44.7; 47.6) |
| Colombia | Medellin | 294 | 280 | 49.8 (49.1; 50.4) | 52.4 (46.5; 58.2) | 21.2 (20.5; 21.9) | 32.9 (32.4; 33.4) |
| Colombia | Barranquilla | 345 | 317 | 51.3 (50.6; 52.0) | 53.0 (47.5; 58.5) | 19.9 (19.3; 20.6) | 31.9 (31.5; 32.4) |
| Colombia | Cartagena | 223 | 212 | 51.3 (50.4; 52.1) | 57.4 (50.7; 64.1) | 28.9 (27.9; 30.0) | 28.5 (27.9; 29.0) |
| Colombia | Manizales | 182 | 172 | 51.5 (50.6; 52.4) | 48.4 (40.9; 55.8) | 15.6 (14.9; 16.3) | 29.4 (28.8; 30.0) |
| Colombia | Valledupar | 131 | 119 | 51.4 (50.4; 52.5) | 48.9 (39.8; 57.9) | 18.7 (17.8; 19.6) | 28.7 (28.0; 29.3) |
| Colombia | Quibdo | 104 | 98 | 52.6 (51.2; 53.9) | 38.5 (28.8; 48.1) | 12.8 (11.9; 13.7) | 28.8 (27.9; 29.6) |
| Colombia | Bogota | 945 | 887 | 49.6 (49.2; 50.0) | 49.0 (45.7; 52.3) | 23.2 (22.8; 23.6) | 27.9 (27.6; 28.1) |
| Colombia | Neiva | 127 | 120 | 48.7 (47.9; 49.5) | 45.7 (36.7; 54.6) | 16.2 (15.4; 17.1) | 29.0 (28.3; 29.7) |
| Colombia | Cucuta | 282 | 266 | 50.6 (49.9; 51.4) | 50.7 (44.7; 56.7) | 12.9 (12.4; 13.4) | 34.3 (33.8; 34.8) |
| Colombia | Armenia | 121 | 110 | 49.0 (47.9; 50.0) | 55.4 (46.0; 64.7) | 23.9 (22.8; 25.1) | 34.3 (33.6; 35.0) |
| Colombia | Pereira | 159 | 152 | 48.5 (47.7; 49.2) | 45.9 (38.0; 53.9) | 19.0 (18.2; 19.9) | 28.7 (28.1; 29.2) |
| Colombia | Bucaramanga | 249 | 233 | 49.9 (49.3; 50.6) | 53.4 (47.0; 59.8) | 24.1 (23.2; 24.9) | 32.1 (31.6; 32.6) |
| Colombia | Sincelejo | 150 | 133 |  | 49.3 (40.8; 57.9) |  |  |
| Colombia | Ibague | 158 | 151 | 50.0 (49.0; 51.0) | 50.6 (42.6; 58.6) | 19.0 (18.1; 20.0) | 30.0 (29.3; 30.6) |
| Colombia | Cali | 196 | 180 | 49.6 (48.8; 50.3) | 54.1 (46.8; 61.4) | 27.2 (26.1; 28.3) | 32.4 (31.7; 33.0) |
| Peru | Chimbote | 307 | 274 | 56.5 (55.9; 57.1) | 57.7 (52.0; 63.3) | 41.5 (40.2; 42.8) | 21.2 (20.7; 21.8) |
| Peru | Huaraz | 141 | 126 | 49.4 (48.2; 50.7) | 50.4 (41.9; 58.9) | 34.9 (33.0; 36.8) | 13.6 (13.0; 14.3) |
| Peru | Arequipa | 747 | 679 | 52.2 (51.7; 52.6) | 52.6 (49.0; 56.3) | 34.8 (34.0; 35.6) | 12.4 (12.2; 12.6) |
| Peru | Ayacucho | 353 | 315 | 55.3 (54.5; 56.1) | 55.8 (50.5; 61.1) | 42.5 (41.2; 43.8) | 12.0 (11.7; 12.4) |
| Peru | Cajamarca | 156 | 141 | 53.0 (51.7; 54.4) | 53.8 (45.9; 61.8) | 35.3 (33.5; 37.1) | 15.2 (14.5; 15.9) |
| Peru | Cusco | 281 | 265 | 52.9 (52.2; 53.6) | 53.7 (47.8; 59.7) | 32.5 (31.3; 33.7) | 15.0 (14.5; 15.4) |
| Peru | Huanuco | 301 | 268 | 48.9 (48.0; 49.7) | 49.2 (43.4; 55.0) | 33.0 (31.7; 34.2) | 15.3 (14.8; 15.8) |
| Peru | Chincha Alta | 359 | 320 | 52.0 (51.4; 52.7) | 53.8 (48.5; 59.0) | 36.6 (35.5; 37.8) | 5.6 (5.4; 5.7) |
| Peru | Ica | 396 | 345 | 50.6 (50.0; 51.2) | 49.5 (44.5; 54.5) | 33.2 (32.1; 34.3) | 10.7 (10.4; 10.9) |
| Peru | Pisco | 156 | 144 | 48.9 (47.9; 49.8) | 48.7 (40.7; 56.7) | 34.4 (32.7; 36.0) | 9.9 (9.5; 10.3) |
| Peru | Huancayo | 441 | 397 | 53.8 (53.2; 54.4) | 56.7 (51.9; 61.5) | 40.3 (39.3; 41.4) | 12.9 (12.6; 13.2) |
| Peru | Chiclayo | 683 | 610 | 56.5 (56.0; 57.0) | 57.7 (53.9; 61.5) | 38.6 (37.7; 39.5) | 17.5 (17.2; 17.8) |
| Peru | Lima | 3278 | 2934 | 53.6 (53.4; 53.8) | 54.7 (52.9; 56.4) | 36.8 (36.5; 37.2) | 16.3 (16.2; 16.4) |
| Peru | Trujillo | 511 | 443 | 57.8 (57.2; 58.4) | 58.7 (54.3; 63.1) | 39.9 (38.8; 41.0) | 20.9 (20.5; 21.4) |
| Peru | Iquitos | 555 | 505 | 59.3 (58.7; 59.8) | 59.8 (55.6; 64.0) | 47.0 (46.0; 48.0) | 18.0 (17.7; 18.4) |
| Peru | Piura | 228 | 211 | 44.6 (43.5; 45.6) | 43.4 (36.9; 50.0) | 36.5 (35.0; 38.0) | 7.3 (7.0; 7.5) |
| Peru | Sullana | 162 | 148 | 37.7 (36.9; 38.6) | 35.8 (28.2; 43.4) | 30.8 (29.2; 32.4) | 4.2 (4.0; 4.3) |
| Peru | Juliaca | 155 | 138 | 43.8 (42.8; 44.8) | 41.9 (34.0; 49.9) | 33.6 (32.0; 35.3) | 8.0 (7.7; 8.3) |
| Peru | Tarapoto | 253 | 220 | 73.3 (72.6; 73.9) | 77.9 (72.6; 83.1) | 54.2 (52.7; 55.7) | 29.8 (29.0; 30.6) |
| Peru | Tacna | 962 | 891 | 50.6 (50.2; 51.0) | 51.7 (48.5; 54.9) | 31.0 (30.4; 31.7) | 15.2 (14.9; 15.4) |
| Peru | Tumbes | 622 | 567 | 37.0 (36.5; 37.4) | 36.5 (32.6; 40.4) | 29.8 (29.0; 30.6) | 5.4 (5.3; 5.5) |
| Peru | Pucallpa | 783 | 734 | 55.0 (54.5; 55.5) | 57.5 (53.9; 61.0) | 36.0 (35.2; 36.8) | 17.4 (17.1; 17.7) |
| El Salvador | San Miguel | 163 | 148 | 46.0 (44.6; 47.4) | 41.7 (34.1; 49.3) | 23.9 (22.6; 25.3) | 13.1 (12.3; 13.8) |
| El Salvador | San Salvador | 1025 | 916 | 48.4 (47.8; 48.9) | 50.6 (47.6; 53.7) | 26.3 (25.9; 26.7) | 13.3 (13.0; 13.6) |
| El Salvador | Santa Ana | 351 | 313 | 46.1 (45.1; 47.0) | 47.9 (42.6; 53.1) | 24.5 (23.6; 25.3) | 12.8 (12.4; 13.3) |
| Guatemala | Guatemala City | 1396 | 1308 | 62.4 (61.9; 62.9) | 64.2 (61.7; 66.7) | 46.1 (45.8; 46.4) | 30.5 (30.0; 30.9) |
| Mexico | Aguascalientes | 580 | 484 | 48.7 (48.1; 49.3) | 49.5 (45.2; 53.8) | 26.3 (25.7; 27.0) | 19.0 (18.5; 19.4) |
| Mexico | La Paz | 262 | 225 | 49.1 (48.2; 50.1) | 51.9 (45.6; 58.2) | 18.2 (17.5; 18.9) | 24.0 (23.3; 24.7) |
| Mexico | Campeche | 173 | 137 | 38.7 (37.5; 39.9) | 35.8 (28.3; 43.4) | 13.3 (12.5; 14.0) | 14.6 (13.9; 15.2) |
| Mexico | Ciudad del Carmen | 127 | 101 | 25.3 (24.1; 26.4) | 18.9 (11.7; 26.1) | 9.8 (9.1; 10.4) | 9.2 (8.6; 9.7) |
| Mexico | Juarez | 103 | 85 | 38.0 (36.5; 39.5) | 40.8 (30.9; 50.6) | 17.9 (16.7; 19.1) | 18.8 (17.8; 19.8) |
| Mexico | Saltillo | 117 | 94 | 40.8 (39.3; 42.3) | 45.3 (35.7; 54.9) | 18.7 (17.4; 20.0) | 19.6 (18.6; 20.7) |
| Mexico | Torreon | 303 | 248 | 31.8 (31.1; 32.4) | 33.0 (27.5; 38.5) | 15.1 (14.5; 15.7) | 13.4 (13.0; 13.9) |
| Mexico | Colima | 182 | 152 | 58.6 (57.5; 59.8) | 60.4 (53.1; 67.7) | 23.7 (22.6; 24.8) | 33.8 (32.7; 34.9) |
| Mexico | Manzanillo | 128 | 104 | 49.5 (48.0; 51.0) | 52.3 (43.1; 61.5) | 17.0 (16.0; 18.0) | 30.3 (29.1; 31.5) |
| Mexico | Tecoman | 105 | 84 | 48.0 (46.7; 49.4) | 51.4 (41.4; 61.4) | 19.9 (18.6; 21.1) | 27.4 (26.2; 28.6) |
| Mexico | Durango | 161 | 130 | 44.0 (42.8; 45.2) | 41.0 (32.9; 49.1) | 18.0 (17.0; 18.9) | 13.7 (13.1; 14.4) |
| Mexico | Acapulco de Juarez | 122 | 100 | 37.5 (36.3; 38.7) | 37.7 (28.8; 46.6) | 16.3 (15.3; 17.4) | 13.4 (12.7; 14.1) |
| Mexico | Celaya | 104 | 85 | 34.4 (33.2; 35.6) | 32.7 (23.0; 42.4) | 19.7 (18.6; 20.8) | 9.5 (9.0; 10.1) |
| Mexico | Leon | 178 | 154 | 44.6 (43.5; 45.6) | 42.7 (35.1; 50.3) | 20.1 (19.2; 21.0) | 15.1 (14.6; 15.7) |
| Mexico | Pachuca de Soto | 151 | 121 | 39.8 (38.6; 40.9) | 39.1 (30.8; 47.3) | 18.8 (17.8; 19.9) | 20.0 (19.1; 20.8) |
| Mexico | Guadalajara | 333 | 277 | 52.9 (51.9; 53.8) | 53.5 (47.9; 59.0) | 18.6 (17.9; 19.3) | 24.2 (23.5; 24.8) |
| Mexico | Mexico City | 645 | 523 | 34.2 (33.7; 34.8) | 38.1 (34.3; 42.0) | 17.3 (16.8; 17.7) | 9.8 (9.6; 10.1) |
| Mexico | Cuautla | 151 | 120 | 42.2 (41.0; 43.4) | 39.1 (30.9; 47.3) | 16.3 (15.4; 17.2) | 19.7 (18.9; 20.6) |
| Mexico | Cuernavaca | 231 | 190 | 37.2 (36.3; 38.2) | 39.8 (33.3; 46.3) | 19.2 (18.4; 20.1) | 15.7 (15.1; 16.3) |
| Mexico | Tepic | 188 | 160 | 45.7 (44.5; 46.9) | 50.5 (43.2; 57.9) | 17.7 (16.8; 18.6) | 24.1 (23.1; 25.1) |
| Mexico | Monterrey | 465 | 369 | 36.1 (35.4; 36.7) | 37.0 (32.4; 41.6) | 14.4 (13.9; 14.9) | 18.7 (18.3; 19.2) |
| Mexico | Oaxaca de Juarez | 103 | 89 | 41.3 (39.9; 42.6) | 40.8 (30.9; 50.7) | 13.0 (12.1; 13.9) | 13.1 (12.4; 13.9) |
| Mexico | Puebla de Zaragoza | 415 | 339 | 34.4 (33.7; 35.1) | 35.4 (30.6; 40.3) | 15.9 (15.4; 16.4) | 11.8 (11.4; 12.1) |
| Mexico | Queretaro | 253 | 216 | 45.2 (44.2; 46.1) | 49.8 (43.4; 56.2) | 20.0 (19.2; 20.7) | 21.0 (20.4; 21.7) |
| Mexico | Cancun | 168 | 148 | 35.4 (34.3; 36.4) | 33.3 (25.8; 40.8) | 13.3 (12.6; 14.0) | 14.6 (14.0; 15.2) |
| Mexico | Culiacan | 110 | 88 | 42.7 (41.3; 44.2) | 40.9 (31.2; 50.6) | 18.2 (17.0; 19.3) | 16.3 (15.4; 17.2) |
| Mexico | San Luis Potosi | 210 | 170 | 35.2 (34.3; 36.1) | 35.7 (28.9; 42.5) | 17.9 (17.0; 18.7) | 12.6 (12.1; 13.1) |
| Mexico | Hermosillo | 119 | 99 | 50.1 (48.9; 51.4) | 52.1 (42.9; 61.3) | 15.9 (15.0; 16.9) | 21.2 (20.3; 22.1) |
| Mexico | Villahermosa | 298 | 265 | 22.4 (21.8; 23.0) | 19.8 (15.1; 24.5) | 7.1 (6.9; 7.4) | 9.5 (9.2; 9.8) |
| Mexico | Tampico | 113 | 91 | 41.8 (40.3; 43.3) | 47.8 (38.2; 57.4) | 14.8 (13.8; 15.9) | 15.9 (15.0; 16.9) |
| Mexico | Tlaxcala | 308 | 266 | 38.9 (38.2; 39.6) | 37.7 (32.0; 43.3) | 20.6 (19.9; 21.4) | 9.9 (9.6; 10.2) |
| Mexico | Merida | 277 | 225 | 51.7 (50.7; 52.7) | 54.5 (48.3; 60.7) | 14.6 (13.9; 15.3) | 32.5 (31.5; 33.4) |
| Mexico | Fresnillo | 156 | 133 | 43.8 (42.8; 44.7) | 43.6 (35.5; 51.7) | 21.4 (20.4; 22.4) | 17.6 (16.9; 18.3) |
| Mexico | Zacatecas | 190 | 164 | 48.6 (47.5; 49.7) | 48.9 (41.5; 56.4) | 26.7 (25.7; 27.8) | 22.9 (22.1; 23.7) |

CI, 95% Confidence Interval; y, years old. Cities with empty indirect estimation were excluded from the sample due to empty data for one or more sex-age strata. Estimated prevalences for population 20 to 69 years old.

**Table S6. Sensitivity analyses: City prevalences of daily fruit intake and daily vegetable intake according to contextual factors.**

|  | **City GDP per capita (2011 USD ppp)** | | |  |
| --- | --- | --- | --- | --- |
| **Daily intake prevalences** | **Tertile 1**  **<$10,800** | **Tertile 2**  **($10,800 to 17,300)** | **Tertile 3**  **(≥$17,300)** | **p for trend** |
| Fruits, median (IQR) | 27.4 (13.5) | 25.7 (12.7) | 30.0 (13.3)^b^ | 0.169 |
| Vegetables, median (IQR) | 22.2 (18.9) | 27.6 (17.9) | 34.3 (24.3)^a,b^ | 0.002 |
| F&V (excludes Mexico), median (IQR)^c^ | 53.8 (15.1) | 57.0 (20.7)^a^ | 69.5 (11.8)^a,b^ | <0.001 |
|  | **City labor force participation** | | |  |
| **Daily intake prevalences** | **Tertile 1 (<60.5%)** | **Tertile 2**  **(60.5 to <64.5%)** | **Tertile 3**  (≥**64.5%)** | **p for trend** |
| Fruits, median (IQR) | 21.4 (15.9) | 26.7 (12.8) | 32.0 (8.0)^a,b^ | 0.002 |
| Vegetables, median (IQR) | 19.6 (18.5) | 23.5 (17.2) | 39.0 (18.3)^a,b^ | <0.001 |
| F&V (excludes Mexico), median (IQR)^c^ | 52.0 (16.0) | 64.0 (18.8)^a^ | 68.4 (12.7)^a,b^ | <0.001 |
|  | **City educational attainment (% with high scool)** | | |  |
| **Daily intake prevalences** | **Tertile 1**  **(<42%)** | **Tertile 2**  **42% to <55%** | **Tertile 3**  **(≥55%)** | **p for trend** |
| Fruits, median (IQR) | 28.5 (11.7) | 21.8 (12.2)^a^ | 31.2 (8.8)^a,b^ | <0.001 |
| Vegetables, median (IQR) | 32.2 (19.0) | 26.2 (20.1) | 28.4 (27.2) | 0.329 |
| F&V (excludes Mexico), median (IQR)^c^ | 64.8 (8.7) | 64.0 (24.0) | 53.7 (21.9)^a^ | 0.120 |
|  | **City WA-Labor force** | | |  |
| **Daily intake prevalences** | **Tertile 1**  **(<-1.10)** | **Tertile 2**  **(-1.10 to <0.85)** | **Tertile 3**  **(≥0.85)** | **p for trend** |
| Fruits, median (IQR) | 19.2 (10.4) | 30.5 (10.6)^a^ | 32.1 (8.0)^a^ | <0.001 |
| Vegetables, median (IQR) | 19.0 (19.0) | 30.0 (19.5)^a^ | 32.8 (21.1)^a^ | 0.007 |
| F&V (excludes Mexico), median (IQR)^c^ | 51.8 (22.4) | 65.5 (11.3)^a^ | 63.8 (19.5)^a^ | 0.038 |
|  | **City Education-WA** | | |  |
| **Daily intake prevalences** | **Tertile 1**  **(<-0.35)** | **Tertile 2**  **(-0.35 to <1.55)** | **Tertile 3**  **(≥1.55)** | **p for trend** |
| Fruits, median (IQR) | 27.9 (13.3) | 24.0 (12.8) | 31.0 (10.9)^a,b^ | 0.008 |
| Vegetables, median (IQR) | 32.2 (21.1) | 24.0 (16.7) | 29.8 (31.3) | 0.262 |
| F&V (excludes Mexico), median (IQR)^c^ | 66.3 (20.6) | 67.2 (10.4) | 51.4 (10.4)^a^ | 0.167 |
|  | **City GII** | | |  |
| **Daily intake prevalences** | **Tertile 1**  **<0.36** | **Tertile 2**  **(0.36 to <0.40)** | **Tertile 3**  **(≥0.40)** | **p for trend** |
| Fruits, median (IQR) | 29.6 (12.1) | 27.6 (12.0) | 19.4 (10.2)^a,b^ | 0.001 |
| Vegetables, median (IQR) | 34.3 (22.6) | 34.3 (13.0) | 20.6 (15.5)^a,b^ | <0.001 |
| F&V (excludes Mexico), median (IQR)^c^ | 66.3 (20.6) | 67.2 (10.4) | 51.4 (10.4)^a,b^ | <0.001 |
|  | **City size (total population x 1,000)** | | |  |
| **Daily intake prevalences** | **Tertile 1**  **(<363)** | **Tertile 2**  **(363 to <1000)** | **Tertile 3**  **(≥1000)** | **p for trend** |
| Fruits, median (IQR) | 29.1 (8.6) | 23.9 (16.5)^a^ | 27.4 (14.2) | 0.181 |
| Vegetables, median (IQR) | 29.8 (26.1) | 26.2 (19.7) | 28.8 (22.0) | 0.767 |
| F&V (excludes Mexico), median (IQR)^c^ | 63.1 (19.8) | 60.0 (20.3) | 64.1 (18.2) | 0.597 |
|  | **Population density (hab/km^2^)** | | |  |
| **Daily intake prevalences** | **Tertile 1**  **(<6,400)** | **Tertile 2**  **(6,400 to 9,600)** | **Tertile 3**  **(≥9,600)** | **p for trend** |
| Fruits, median (IQR) | 27.9 (13.8) | 26.7 (15.3) | 29.1 (13.3)^b^ | 0.092 |
| Vegetables, median (IQR) | 33.3 (18.2) | 23.9 (27.5)^a^ | 28.5 (18.8)^a^ | 0.003 |
| F&V (excludes Mexico), median (IQR)^c^ | 68.4 (9.1) | 67.3 (18.2) | 51.3 (7.3)^a,b^ | <0.001 |
|  |  |  |  |  |
|  | **Climate Zone** | | |  |
| **Daily intake prevalences** | **Tropical** | **Arid** | **Temperate** | **p for trend** |
| Fruits, median (IQR) | 24.2 (13.3) | 27.6 (15.1)^a^ | 30.8 (6.8)^a^ | 0.004 |
| Vegetables, median (IQR) | 28.7 (15.7) | 20.4 (18.4) | 34.7 (22.4)^a,b^ | 0.003 |
| F&V (excludes Mexico), median (IQR)^c^ | 56.9 (19.5) | 58.1 (15.7) | 68.7 (15.6)^a,b^ | 0.011 |

IQR, Interquartile Range, WA, Women Achievement score, GDP, gross domestic product, ppp, purchase power parity, GII, Gender inequality index). ^a^p<0.05 from tertile 1; ^b^p<0.05 from tertile 2. ^c^Different tertile values for city variables when excluding cities from Mexico (GDP tertiles: 1) <10,000; 2) 10,000 to <16,900; 3)≥16,900. Labor force: 1) <61%; 2) 61% to 66.3%; 3)>66.3%. Educational attainment tertiles: 1)<42%; 2)42% to <59%; 3)≥59%. Labor WA tertiles: 1) <0; 2)0 to ≤1.00; 3)>1.00. Education WA tertiles: 1) <0.20 2)0.20 to <2.17; 3)≥2.17. GII tertiles:1) <0.36; 2)0.36 to ≤0.39; 3)>0.39. City size (x 1,000 hab) tertiles: 1)<315; 2)315 to <992; 3) ≥992. Population density tertile:1)<6,600; 2)6,600 to 10,800; 3)>10,800). Prevalences of daily fruit and vegetable intake are treated as a continuous variable, hence, they are expressed as the median (IQR) city prevalences within each tertile or category. Women Achievement score tertiles indicate cities with higher women autonomy (WA-Labor force includes proportion of female labor force participation and formal marriage in women 15 – 17 years old) and female educational attainment scores (WA-Education includes proportion women ≥25 years old with high school and college education). Higher tertile GII indicate cities with higher gender inequality (includes labor force, political participation and educational attainment in relation to males). Cities with polar climate zone were excluded because of sample size (n=2).
